# Mixed methods evaluation of the COVID-19 changes to the WIC cash-value benefit for fruits and vegetables

**DOI:** 10.3389/fpubh.2024.1371697

**Published:** 2024-04-29

**Authors:** Allison M. Nitto, Mayra Crespo-Bellido, Jackie Yenerall, Elizabeth T. Anderson Steeves, Sarah K. Kersten, Daniele Vest, Jennie L. Hill

**Affiliations:** ^1^Gretchen Swanson Center for Nutrition, Omaha, NE, United States; ^2^Department of Agricultural and Resource Economics, University of Tennessee, Knoxville, TN, United States; ^3^Population Health Sciences, University of Utah, Salt Lake City, UT, United States

**Keywords:** WIC, policy evaluation, COVID-19, nutrition assistance programs, implementation science

## Abstract

**Introduction:**

Recent cash-value benefit (CVB) increases are a positive development to help increase WIC participant fruits and vegetables (FV) access. Little is known about the impacts of the CVB changes on FV redemptions or about implementation successes and challenges among WIC State and local agencies. This mixed method study aimed to evaluate (a) the CVB changes’ impact on FV access among WIC child participants measured by CVB redemption rates, (b) facilitators and barriers to CVB changes’ implementation, and (c) differences in FV redemption and facilitators and barriers by race/ethnicity.

**Methods:**

We requested redemption data from all 89 State agencies for April 2020 to September 2022 and utilized descriptive statistics, interrupted time series analysis (ITS), and generalized linear regression analysis. Additionally, we recruited State agencies, local agencies, and caregivers across the U.S. for interviews and used rapid qualitative analysis to find emerging themes anchored in policy evaluation and implementation science frameworks.

**Results:**

We received redemption data from 27 State agencies and interviewed 23 State agencies, 61 local agencies, and 76 caregivers of child WIC participants. CVB monthly redemptions increased at $35/child/month compared to $9/child/month; however, adjusted ITS analyses found a decrease in redemption rates at $35/child/month. The decrease was not significant when the transition/first implementation month was excluded with rates progressively increasing over time. Differences were found among racial/ethnic groups, with lower redemption rates observed for non-Hispanic Black caregivers. Overall, WIC caregivers reported high satisfaction and utilization at the $35/child/month. The frequent and quick turnaround CVB changes strained WIC agency resources with agencies serving higher caseloads of diverse racial and ethnic populations experiencing greater issues with implementing the CVB changes.

**Conclusion:**

Despite implementation challenges, the increased CVB shows promise to improve WIC participant FV access and satisfaction with WIC. WIC agencies need adequate lead time to update the CVB amounts, and resources and support to help ensure equitable distribution and utilization of the FV benefits.

## 1 Introduction

In 2022, over 6.2 million pregnant, postpartum, and breastfeeding women and children up to age five were served by the Special Supplemental Nutrition Program for Women, Infants, and Children (WIC) (1). WIC is administered by the U.S. Department of Agriculture’s Food and Nutrition Service (FNS) and through 89 WIC agencies at the State level, including 50 States and the District of Columbia, 33 Indian Tribal Organizations (ITOs), and five territories (2). Participation in WIC brings several health benefits, including improvement in child feeding practices and increases in adequate growth/weight status and cognitive development for infant and child participants (3, 4). The WIC food package is a pillar of WIC services as it provides essential food to support a healthy, well-balanced diet (5). The cash-value benefit (CVB) is part of the food package, which allocates a monthly dollar amount to spend on fruits and vegetables (FVs) (5). Research shows that exposure to a variety of healthy foods promotes the development of healthy food preferences early and that continues later in life (6). Thus, the CVB plays a critical role in providing millions of WIC participants nationwide with healthy foods that can address key nutritional gaps through early childhood (7). However, the CVB monthly amount must be sufficient to make notable impacts on WIC participants’ diets (8, 9).

Policymakers addressed potential food and nutrition security crises during the COVID-19 pandemic by leveraging existing programs and implementing changes that included enhancing federal food assistance benefits. For WIC, until 2021, the CVB amount was $9/child/month; however, on March 11, 2021, the American Rescue Plan Act of 2021 (ARPA) offered WIC State agencies the option to increase the CVB to $35/child/month for up to 4 months (10, 11). Four WIC State agencies did not opt into the $35/child/month increase. In October 2021, the continued resolution (CR) required that all WIC State agencies change the CVB to $24/child/month and subsequent increases to account for inflation occurred in October 2022 ($25/child/month) and October 2023 ($26/child/month) (12). The increases to the CVB dollar amount align with nutrition and medical expert recommendations on the amount of FVs needed to support a healthy diet (13). Recent studies have shown that CVB changes to both $35/child/month and $24/child/month compared to the $9/child/month resulted in increased consumption of FVs (14), a greater diversity of FVs consumed (15, 16), and economic benefits of investing in local retailers through increased purchasing of FVs (17). Still, CVB policy changes and their implementation occurred during unprecedented tumultuous times when WIC agencies had multiple competing priorities (e.g., shift to remote services, national formula recall). Therefore, it is critical to evaluate how the implementation of these CVB policy changes impacted children’s access to FVs.

Prior scholarship in public policy evaluation demonstrates that policy enactment does not ensure that the policy will be implemented as intended (18). There are known barriers that can potentially impact WIC agencies’ ability to implement policy-driven programmatic changes. For example, the 2023 WIC Technology Landscape Report found technology disparities as WIC agencies upgraded their systems to accommodate new electronic benefit transfer mandatory policies. These disparities were attributed to funding, staff capacity, technical expertise, and other competing priorities among others. The present mixed methods evaluation of the CVB policy changes serves several research aims: (a) identify the degree to which the implemented policy achieved its intended outcomes (19) of increasing access to FVs among young children (up to 5 years of age) participating in WIC, measured by WIC FV redemption rates; (b) enhance future policy implementation effectiveness (e.g., WIC food package changes) by identifying barriers and facilitators to policy-driven programmatic change based on qualitative interviews with WIC State agencies and local agencies and caregivers of child participants; and (c) identify any differences in redemptions by WIC participant race, ethnicity, and urbanicity and strategies to foster equity and reduce inconsistencies in service delivery across WIC sites.

## 2 Methods

### 2.1 Design and frameworks

**Figure 1:**
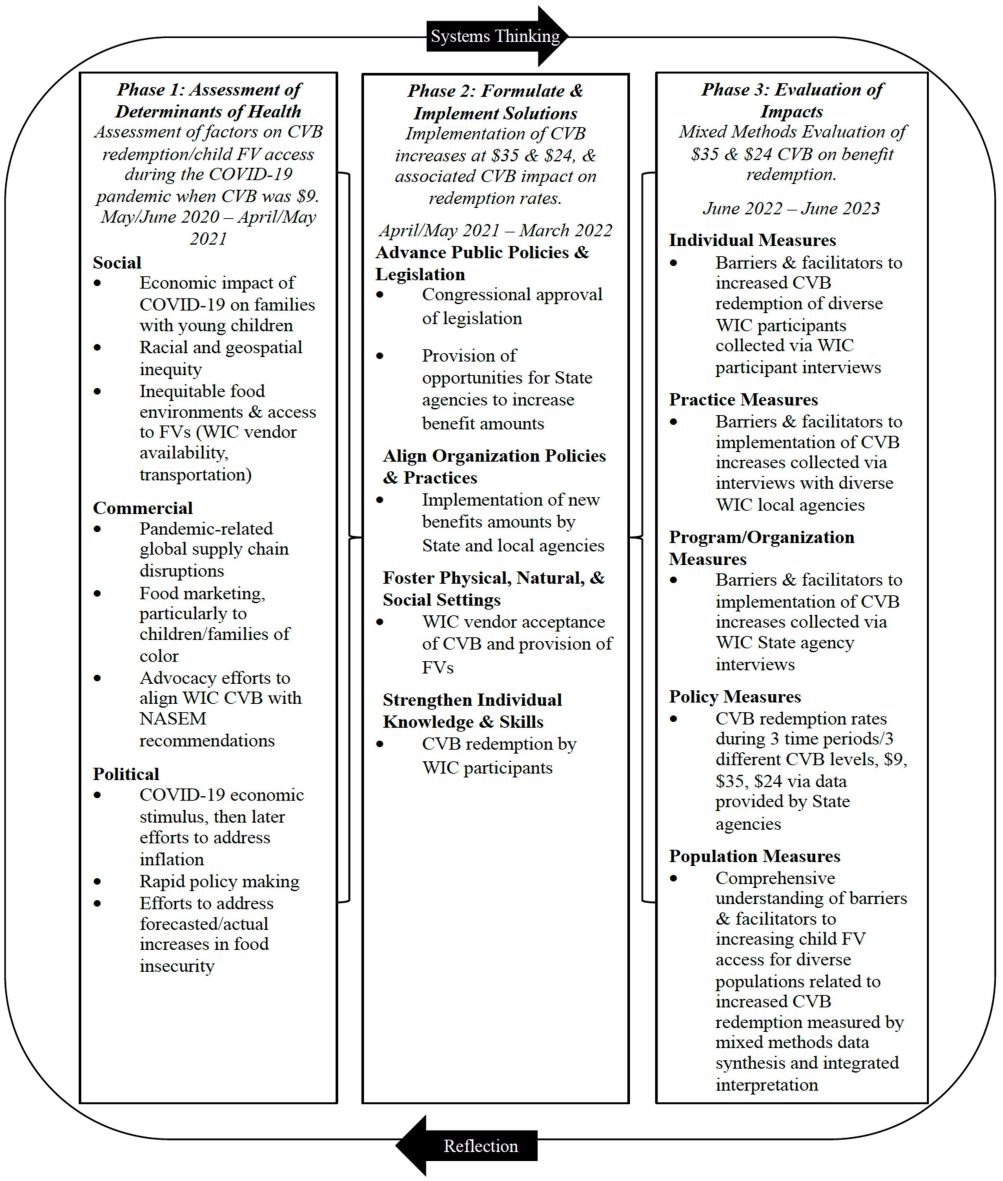
Mapping of research design aligned with the I + PSE Framework.

This study used a convergent parallel mixed methods design (20) that included quantitative data collection and analysis of WIC State agency administrative data and qualitative data collection and analysis of interviews with WIC State and local agency staff and caregivers of child participants. The data was merged and the findings of each strand were triangulated at the interpretation phase (21). Two implementation and evaluation frameworks were used in the study design. First, the study used the Individual plus Policy, System, and Environmental (I + PSE) Conceptual Framework for Action, which uses a three-phase model [with phases including: (1) assess determinants of health, (2) formulate and implement solutions, (3) evaluate impacts] to address challenges and identify multidimensional strategies to improve nutrition and health using systems thinking and iterative reflection (22). For this study, policy processes related to the WIC CVB were considered the determinant of health, and new policies related to increasing the CVB amount were implemented to help address the negative impact of COVID-19, including the rising cost of food prices, on FV consumption of WIC participants. Thus, this study evaluated the impacts of the changes at the individual, practice, program, organization, policy, and population levels (Figure 1).

The qualitative interviews with WIC State and local agencies were grounded in the integrated Promoting Action on Research Implementation in Health Services (i-PARIHS) framework (23, 24) with adaptations to align with Phase 3 of the I + PSE. i-PARIHS is a well-established implementation science framework developed to identify factors related to successful implementation in real-world settings and helped answer a fundamental research question as to why implementation of the CVB changes were or were not successful. The i-PARIHS framework has four core elements—evidence, recipients, context, and facilitation—and each of these has multiple components that were aligned to I + PSE Phase 3 evaluation impacts. Variables under context within i-PARIHS, including leadership support, culture, and receptivity to change, are aligned with agility (practice), leadership capacity (strategic management), and adaptive culture (organization). The i-PARIHS elements relate to organizational readiness for adoption, and barriers to and facilitators for successful implementation and sustainability of new initiatives are also adaptable for the I + PSE.

### 2.2 Data collection

#### 2.2.1 WIC administrative data

Data was requested from all 89 WIC State agencies from April 2020 to September 2022, which includes baseline data at $9/child/month, policy change 1 data (increase to $35/child/month), and policy change 2 data (decrease to $24/child/month). We used a previous registry of WIC State agency directors who were contacted up to five times throughout the data collection period. Initial outreach began with an email, followed by three reminder emails, before transitioning to phone calls. The data collection period was October 2022 – April 2023. We tracked responses by USDA FNS Region (25) (Mid-Atlantic, Midwest, Mountain Plains, Northeast, Southeast, Southwest, and Western), WIC State agency type (State, Indian Tribal Organization [ITO]/Territory), and WIC participant caseload (<10,000; 10,000 – 75,000; >75,000) and tailored our recruitment strategies to encourage responses across the different WIC State agency characteristics (e.g., personalized emails communicating response rates to encourage recruitment from ITO agencies). All data was collected at the state-month observation level and included household data, which covered the total count of child participants, total count of adult participants (pregnant, postpartum non-breastfeeding, and breastfeeding certification categories), and total count of WIC households; sum of WIC household size (i.e., participants and non-participants) for all households; and total count of WIC households by racial/ethnic group and county. We also requested the sum of all CVBs issued and redeemed in each State in each month, overall and by racial/ethnic group and county in addition to household CVB redemption rates (calculated by dividing CVB redeemed by CVB issued and multiplying by 100). WIC State agencies were offered $200 for the time needed to provide the administrative data with the option to donate the funds to a charity.

#### 2.2.2 Qualitative interviews

The semi-structured interview guides were developed to align with i-PARIHS and tailored based on the type of respondent: (1) WIC State agency directors, (2) WIC local agency directors and nutritionists, and (3) caregivers of WIC child participants. State agencies were asked about their role and responsibilities related to the CVB implementation, any procedures and technology changes implemented, guidance and communication from federal agencies, guidance and communication provided to local agencies, and challenges and successes during the CVB changes. Local agency staff were asked about procedures needed to implement CVB changes, guidance and communication from State agencies, education and promotion of CVB changes provided to WIC participants, and challenges and successes during the CVB changes. Caregivers of WIC child participants were asked about their awareness, utilization, and satisfaction with each CVB amount (e.g., $9, $35, $24 per child per month). All interviews were offered in both English and Spanish. The interview guides also were reviewed by an external expert advisory panel comprised of two WIC State agency staff, two local agency staff, and two WIC participants.

Similar to the WIC State agency administrative data, we used a previous registry of WIC State agency directors and invited all 89 WIC State agency directors to participate in the telephone interviews. Purposive and convenience sampling was used for the WIC local agency and caregivers of WIC participant interviews. For the WIC local agency interviews, we asked WIC State agencies to provide contact information for up to five local WIC directors from their State to participate in the telephone interviews. We also asked WIC State agencies to provide contact information for both rural and urban agencies and agencies that serve varying caseload levels. When contacting local agency directors to participate in the interviews, we asked directors for names of up to three WIC nutritionists at their agency who could also participate in the interview due to their direct interactions with WIC participants during the CVB changes. We received contact information for approximately 111 local agency staff (90 were contacted). WIC State agency and local agency staff were contacted up to five times throughout the data collection period of October 2022 – February 2023. Initial outreach began with an email, followed by a reminder email, before transitioning to phone calls. If phone numbers were available and respondents did not answer, voicemails were left. Contact attempts varied in days and times to accommodate potential respondent preferences. State and local interviews lasted approximately 45 to 60 min and respondents were given the option to accept a $50 gift card or donate $50 to a charity. During data collection, we tracked responses by interviewee characteristics and tailored our recruitment strategies to encourage responses across various agencies. For State agencies, we tracked responses by USDA FNS Region, WIC State agency type, and WIC participant caseload, and for local agencies, by USDA FNS Region, WIC participant caseload (<750; 750–1,999; 2,000 – 4,499; >4,500 participants), and urbanicity (rural vs. urban).

When reaching out to local agencies to participate in the interviews, we also asked if they would promote the WIC caregiver interview through their preferred communication channels (e.g., social media, texting, flyers in the clinic, etc.). We provided local agencies with a digital flyer and offered to mail a hard copy flyer to post in their clinics. The flyer included the study telephone number and email where interested respondents could contact the research team to complete the interview. The interviews lasted approximately 30 min, and respondents were given the option to accept a $30 gift card or donate $30 to a charity. Interviews were conducted from November 2022 – February 2023. To help increase the diversity of responses, we tracked responses by FNS Region, race, ethnicity, and rural/urban, and tailored our recruitment strategies to ensure responses across the different characteristics similar to the State and local agency interviews.

WIC State and local agency and caregiver interviews were conducted by five trained research staff. Prior to starting the interviews, respondents were informed that the interview was voluntary and that they could skip any question and end the interview at any time. During interviews, researchers took detailed notes. Interviews were audio recorded with permission from the respondents and transcribed verbatim by Rev. (Austin, Texas, U.S.), a third-party transcription service. Researchers checked all transcripts for quality assurance and removed any identifiable information from the transcripts. The University of Nebraska Medical Center Office of Regulatory Affairs determined the study to be exempt from human subjects’ review (Exemption #0574-22-EX).

### 2.3 Data analysis

#### 2.3.1 WIC administrative data

The data was cleaned, standardized, and analyzed using SAS version 9.4 (Raleigh, North Carolina). The data was organized into four distinct datasets: overall CVB issuance and redemption data, data by race/ethnicity category, data by county (urbanicity), and data by FV subcategory. Quality control of the data was conducted by trained staff, during which inconsistencies, illogical data points, outliers, and missing values were identified and addressed. State agency staff were subsequently contacted to clarify any discrepancies or issues in the data provided. An indicator denoting the months between the pre- and post-intervention periods (i.e., $35/child/month and $24/child/month) was created.

Descriptive statistics for the WIC State agencies that submitted data were conducted, including percentages of responses by USDA FNS Region, average caseload, and number of agencies that supplied county, race/ethnicity, and FV subcategory data. Differences in these descriptive characteristics among State agencies were assessed using t-tests and Fisher’s exact tests. A naive approach juxtaposed the average redemption rates before and after each policy change using data from April 2020 to September 2022. An interrupted time series (ITS) analysis using data from April 2020 to September 2021 was employed to examine the influence of the change to $35/child/month on CVB redemption rates. The ITS was limited to $35/child/month due to the nature of the administrative data and the two policies. Pregnant and breastfeeding women also receive CVBs. Only for the $35/child/month did the change in policy represent an increase in CVB for all households. For the second policy change, CVB benefits further increased for pregnant and breastfeeding participants but declined to $24/child/month for children. Thus, the change in household CVB benefits due to the second policy change would depend on the composition of participants in the household. However, the administrative data contains redemption rates for all participating households in a State, and it is not possible to isolate the redemption rates for children. To best identify the influence of an increase in CVB benefits on redemption rates we limit our ITS to $35/child/month and use PROC SURVEYREG to explore the relationship between CVB issuance and redemption during the entire observation period.

The ITS model for $35/child/month is specified as follows:


$$\begin{array}{l} CVB\;\mathrm{Redemption}\;\mathrm{Rat}e_{st}=\beta_{0}+\beta_{1}\left(Time_{t}\right)+\beta_{2}\left(\mathrm{Policy}\;\mathrm{Chang}e_{st}\right)\\ +\beta_{3}\left(\mathrm{Time}*\mathrm{PolicyChang}e_{st}\right)+\beta_{cov}\left(\mathrm{Covariate}s_{st}\right)+\epsilon \end{array}$$

Where: CVB Redemption Rate is the dependent variable, representing the monthly total CVB redemption rate for a given State *s* in month *t*. β0: the intercept, representing the expected CVB redemption rate in the absence of any intervention. Time: a continuous variable representing the time (in months) from the start of the pre-intervention period to the end of the post-intervention period. β1: the coefficient for Time, representing the expected change in CVB redemption over time in the absence of any intervention. Policy Change: a binary variable, taking the value of 1 for all months after the policy intervention and 0 otherwise. β2: the coefficient for Policy Change, representing the average difference in CVB redemption rates in the post and pre-policy time periods. Time*Policy Change: an interaction term between Time and Policy Change, allowing for a change in the slope of the trend line in the post-intervention period. β3: the coefficient for Time*Policy Change, representing the change in the slope of the trend line in the post-intervention period. βcov: the coefficient vector for each covariate in the adjusted model. Covariates: the following covariates were used in the adjusted regression models: number of adult participants, number of child participants, and State indicators (i.e., dummy variables to indicate the state of origin for the observation). ε: the error term, representing the random variation in CVB redemption not accounted for by the model. The interrupted time series model was estimated using data from April 2020 to September 2021 and a linear regression with cluster robust errors at the State level. An unadjusted interrupted time series analysis by domain (i.e., demographic characteristics) was also used to explore the differences in CVB redemption rates over pre-implementation and after the CVB changes were implemented for individuals in rural versus urban counties and across different racial/ethnic groups.

The association between CVB issuance and redemption from April 2020 to September 2022 was established through generalized linear regression model (using PROC GLM) specified as follows:


$$\begin{array}{l} CVB\;\mathrm{Dollars}\;\mathrm{Redeeme}d_{\mathrm{St}}=\beta_{0}+\beta_{1}\left(CVB\;\mathrm{Dollars}\;\mathrm{Issue}d_{st}\right)\\ +\beta_{\text{cov}}\left(\mathrm{Covariate}s_{\mathrm{St}}\right)+\epsilon \end{array}$$

Where: CVB dollars redeemed is the dependent variable, representing the monthly total CVB dollars for a given State *s* in month *t*. β0: the intercept, representing the expected CVB dollars redeemed in the absence of any intervention. CVB dollars issued: a continuous variable representing dollars issued for CVB each month. β1: the coefficient for CVB dollars issued, representing the expected change in CVB dollars redeemed for every additional dollar issued. Covariates: the following covariates were used in the adjusted generalized linear regression models: number of adult participants, number of child participants, and State indicators. βcov: the coefficient vector for each covariate in the adjusted model.

#### 2.3.2 Qualitative interviews

This study used a rapid qualitative analysis (26), characterized by its efficiency, participatory nature, team-based collaborative efforts, and iterative progression until theoretical saturation. The team began by crafting specific codes from interview questions, anchored in the i-PARIHS and I + PSE frameworks. A code matrix, which underwent two rounds of piloting for refinement, was developed to summarize these insights and capture illustrative quotes. A review of the entire transcript, followed by an examination of codes and their definitions, was conducted before coding. NVivo 12 software (Burlington, Massachusetts, U.S.) was utilized for coding text within the transcripts. Coders compiled summaries into a matrix format for the rapid identification of patterns, similarities, and differences across respondents. The team then reviewed the summaries to identify overarching themes and subthemes aligned with i-PARIHS. We also conducted a sub-analysis to identify implementation challenges among local agencies with high racial diversity identified as those with greater proportions of Black, Indigenous, and people of color (BIPOC) participants compared to the national racial and ethnic distribution (27).

#### 2.3.3 Mixed method analysis

The mixed method analysis of this convergent parallel study design occurred at the interpretation phase (21). The quantitative tables were triangulated with the themes in the qualitative matrices and areas of convergence and divergence were explored. For example, the administrative data analysis showed how participants are redeeming CVB, the breakdown of redemption rates among racial/ethnic groups, and urbanicity. The qualitative analysis then refined and explored factors related to implementation by WIC agencies that were not readily identified from redemption data alone. The mixed method interpretation helped answer important questions about how the CVB increase was implemented, for whom it worked, and if it did not, why not? Importantly, if the CVB increase did not impact redemption data, or did not increase it equally among all groups, the qualitative phase provided insights into the potential barriers and facilitators to implementation. The mixed method interpretation is incorporated in the discussion section below.

## 3 Results

### 3.1 Respondent characteristics

#### 3.1.1 WIC state agencies

Table 1 shows the distribution of characteristics of the WIC State agencies that provided administrative data and participated in the interviews. Overall, 27 WIC State agencies provided administrative data and 23 WIC State agencies participated in the interviews. Half of the WIC State agencies (*n* = 13) that provided administrative data also participated in the interviews. A higher number of responses were received from agencies in the Western Region, State agencies (vs. ITO/Territory), and medium-sized agencies (i.e., participant caseload of 10,000-75,000). We did not receive administrative data from the four WIC State agencies that did not opt into the first CVB increase of $35/child/month; however, we completed an interview with one WIC State agency that did not opt into the first increase. Please see Supplementary Table S1 that includes the characteristics of the 27 WIC State agencies that provided administrative data and the characteristics of all 89 WIC State agencies.

**Table 1:** Characteristics of WIC state agencies that provided administrative data (*n* = 27) and participated in interviews (*n* = 23).

State agency characteristics	Administrative data	Interviews
FNS Region		
Mid-Atlantic	1 (3.7)	2 (8.7)
Midwest	5 (18.5)	3 (13.0)
Mountain Plains	3 (11.1)	3 (13.0)
Northeast	3 (11.1)	3 (13.0)
Southeast	2 (7.4)	3 (13.0)
Southwest	5 (18.5)	3 (13.0)
Western	8 (29.6)	6 (26.1)
Type of agency		
State	23 (85.2)	18 (78.3)
ITO/Territory	4 (14.8)	5 (21.7)
Participant caseload		
<10,000	5 (18.5)	4 (17.4)
10,000-75,000	15 (55.6)	15 (65.2)
>75,000	7 (25.9)	4 (17.4)
Opted into $35/child/month		
Yes	27 (100.0)	22 (95.7)
No	0 (0.0)	1 (4.3)
Type of data^*^		
Overall data only	15 (55.6)	NA
County-level	12 (44.4)	NA
Race/ethnicity	11 (40.7)	NA
FV subcategories	8 (29.6)	NA

^*^Not mutually exclusive. FNS, Food and Nutrition Service; CVB, cash value benefit; ITO, Indian Tribal Organization; FV, fruit and vegetable.

#### 3.1.2 WIC local agencies

Table 2 summarizes the characteristics of the 61 WIC local agencies that participated in the interviews. A higher percentage of local agencies were part of States (83.6%), had caseloads greater than 4,500 participants (32.8%), and were in urban areas (63.9%). The type of staff responding to the survey included local agency directors (49.2%), nutritionists (37.7%), and other key staff, such as breastfeeding coordinators, vendor managers, and nutrition educators (13.1%).

**Table 2:** Characteristics of WIC local agencies that participated in interviews (*n* = 61).

Local agency characteristics	*n* (%)
FNS Region	
Mid-Atlantic	6 (9.8)
Midwest	14 (23.0)
Mountain Plains	7 (11.5)
Northeast	8 (13.1)
Southeast	13 (21.3)
Southwest	6 (9.8)
Western	7 (11.5)
WIC state agency type	
State	51 (83.6)
ITO/Territory	10 (16.4)
Participant caseload	
<750	13 (21.3)
750–1,999	13 (21.3)
2,000-4,499	15 (24.6)
>4,500	20 (32.8)
Urbanicity	
Rural	22 (36.1)
Urban	39 (63.9)
Respondent staff role	
Local agency director	30 (49.2)
Nutritionist	23 (37.7)
Other	8 (13.1)

FNS, Food and Nutrition Service; ITO, Indian Tribal Organization.

#### 3.1.3 WIC participants

Characteristics of the 76 WIC participants who participated in the interviews are highlighted in Table 3. Approximately half of the participants identified as White, one-fourth as African American or Black, and one-fifth as American Indian/Alaska Native, other race, or multiracial. Nearly one-third of participants identified as Hispanic/Latino. Participants residing in rural or urban areas were close to an even split – 46.0 and 54.0%, respectively.

**Table 3:** Characteristics of WIC participants that participated in interviews (*n* = 76).

WIC participant characteristics	*n* (%)
FNS Region	
Mid-Atlantic	10 (13.2)
Midwest	19 (25.0)
Mountain Plains	15 (19.7)
Northeast	10 (13.2)
Southeast	10 (13.2)
Southwest	10 (13.2)
Western	2 (2.6)
Race	
African American/Black	18 (23.7)
American Indian/Alaska Native	8 (10.5)
White	36 (47.4)
Other Race/Multi-racial	8 (10.5)
Did not report	6 (7.9)
Ethnicity	
Hispanic/Latino	22 (28.9)
Not Hispanic/Latino	54 (71.1)
Urbanicity	
Rural	35 (46.0)
Urban	41 (54.0)

FNS, Food and Nutrition Service.

### 3.2 WIC administrative data

#### 3.2.1 CVB redemption rates

**Figure 2:**
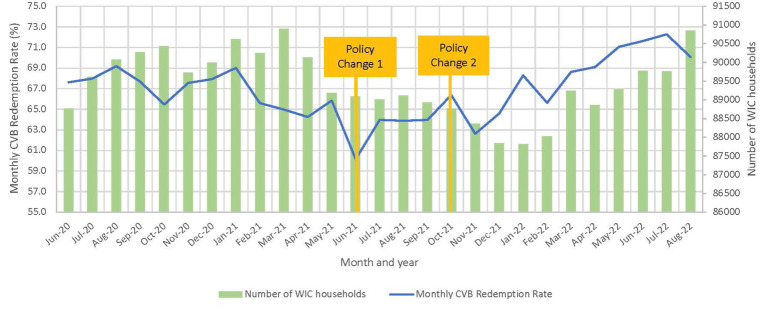
Overall state-level monthly CVB redemption rate and average number of WIC households (*n* = 23 State agency datasets). State agencies that implemented Policy Change 1 (PC1) in May were excluded.

The overall redemption rate decreased from 67.2 to 62.8% at $35/child/month and then increased to 67.9% when changed to $24/child/month (Table 4). Further investigation into the 4 months at $35/child/month identified a sharp decrease in the redemption rate to approximately 60.2% during the first month of implementing the $35/child/month, and then the rate increased in the second month and stabilized for the rest of the policy period to 64.0% (Figure 2). For this reason, the first month of $35/child/month was considered a ‘transition month’ and was dropped from subsequent analysis in the ITS model. With the transition month removed, the coefficient is no longer significant and in a positive trend (Table 5). Additionally, Figure 2 shows that despite the lack of significant change in redemption rates evidenced by the ITS, redemption rates increased over time during the $24/child participant period.

**Table 4:** Average issuance, redemption, and redemption rates during baseline, first policy change, and second policy change; April 2020 to September 2022 (*n* = 810 monthly observations; 30 months times the 27 responding WIC State agencies).

	Pre-implementation	Policy change 1	Policy change 2
Dollar amount issued per child per month	$9	$35	$24
Implementation dates^*^	Apr 2020 – Apr 2021	May 2021 – Aug 2021	Oct 2021 – Sep 2022
		Jun 2021 – Sep 2021	
Monthly CVB issuance [mean (95% CI)]	$926,163.15 ($758,462.45–$1,093,863.85)	$3,345,933.54 ($2,206,417.80–$4,485,449.28)	$2,861,296.29 ($2,306,143.10–$3,416,449.49)
Monthly CVB redemption [mean (95% CI)]	$609,464.48 ($503,425.83–$715,503.14)	$2,073,513.95 ($ 1,359,221.98-$2,787,805.92)	$1,952,969.16 ($1,580,331.17–$2,325,607.15)
Average monthly redemption rate [mean (95% CI)]	67.2 (66.1–68.3)	62.8 (60.9–64.8)	67.9 (66.7–69.1)

^*^WIC State agencies could choose when to implement up to 4 months of the first policy change with agencies starting in May or June 2021; Policy change 1: American Rescue Plan Act (from May to Aug 2021 or Jun to Sep 2021); Policy change 2: Continuing Resolution (from Oct 2021 to Sep 2022). CI, confidence interval; CVB, cash value benefit.

**Table 5:** Unadjusted and adjusted interrupted time series regression analysis of CVB redemption rate before and after first policy increase to a CVB of $35/child/month; April 2020 to September 2021 (*n* = 459 monthly observations; 17 months times the 27 responding WIC State agencies).

Coefficient	Variable	Crude				Adjusted			
		Estimate	SE	*t*-statistic	*p*-value	Estimate	SE	*t*-statistic	*p*-value
β0	Intercept	69.07	2.82	24.48	<0.0001	50.24	4.54	11.04	<0.0001
β1	Time	−0.27	0.16	−1.72	0.09	−0.14	0.1	−1.45	0.16
β2	Policy change	19.53	19.05	1.03	0.31	3.44	3.21	1.07	0.3
β3	Time* policy change	−1.19	1.11	−1.08	0.29	−0.27	0.2	−1.36	0.18

Adjusted for adult participants, child participants, and State indicators. Robust standard errors were clustered at the State level. Removed transition month (i.e., first month of implemented increased CVB). CVB, cash value benefit; SE, standard error.

When analyzing redemption rates by race/ethnicity, redemption rates for all groups decreased when the $35/child/month went into effect, and then an increase was observed with the change to $24/child/month (Table 6). When comparing across race/ethnicity groups, non-Hispanic African American/Black WIC families had the lowest redemption rates whereas non-Hispanic Asian families had the highest redemption rates. When conducting the interrupted time series regression sensitivity analysis of CVB redemption rates by demographic characteristics with the transition month removed, no significant differences in CVB redemption rates by race/ethnicity were observed (Table 7). Lastly, no differences in redemption rates were observed between participants located in rural versus urban areas (Table 7).

**Table 6:** Redemption rates of WIC households served monthly by race/ethnicity.

	NH White	NH Black	NH Asian	Hispanic	NH other race*
Monthly average of households served	231,514	125,581	43,061	596,047	75,077
Monthly CVB redemption rate [mean (95% CI)]					
Pre-implementation	70.3 (67.6–73.0)	66.2 (64.0–68.4)	81.1 (79.5–82.7)	74.8 (73.2–76.4)	69.3 (67.9–70.6)
Policy change 1	64.6 (59.7–69.5)	57.7 (53.6–61.8)	77.7 (74.3–81.0)	69.3 (66.5–72.1)	64.9 (61.9–67.9)
Policy change 2	70.8 (68.2–73.4)	65.2 (63.0–67.4)	82.1 (80.3–83.8)	76.1 (74.2–77.9)	71.2 (69.5–72.9)

^*^States capture racial and ethnic information in different formats. NH Other Race includes multiracial, NH Native American, and “other” categories. Policy change 1: $35/child/month; American Rescue Plan Act (from May to Aug 2021 or Jun to Sep 2021); Policy change 2: $24/child/month; Continuing resolution (from Oct 2021 to Sep 2022). CI, confidence interval; CVB, cash value benefit; NH, Non-Hispanic.

**Table 7:** Interrupted time series regression analysis of CVB redemption rates by demographic characteristics before and after First policy increase to a CVB of $35/child/month; April 2020 to September 2021.

Characteristic	β0			β1			β2			β3		
	Intercept			Time			Intervention			Time*intervention		
	Estimate	SE	*t*-statistic	Estimate	SE	*t*-statistic	Estimate	SE	*t*-statistic	Estimate	SE	*t*-statistic
Overall (*n* = 7,832)	66.92	4.17	16.04	−0.48	0.33	−1.42	−30.45	36.58	−0.83	1.97	1.98	1.00
County urbanicity												
Urban (*n* = 3,757)	70.31^*^	3.92	17.96	−0.69^*^	0.29	−2.35	−16.40	39.20	−0.42	1.24	2.15	0.58
Rural (*n* = 3,918)	63.66^*^	4.68	13.61	−0.30	0.38	−0.80	−44.02	31.5	−1.40	2.69	1.68	1.61
Race/ethnicity												
NH White (*n* = 176)	70.27^*^	4.03	17.45	0.01	0.58	0.01	60.25	74.18	0.81	−3.88	4.66	−0.83
NH Black (*n* = 159)	71.37^*^	4.09	17.45	−0.68^*^	0.23	−2.87	−76.45	38.76	−1.97	4.51	2.23	2.02
NH Asian (*n* = 176)	83.20^*^	2.52	33.04	−0.28	0.26	−1.11	11.89	28.78	0.41	−0.66	1.68	−0.39
NH Other^**^ (*n* = 229)	69.64^*^	2.48	28.18	−0.05	0.18	−0.29	−10.12	22.48	−0.45	0.51	1.30	0.39
Hispanic (*n* = 176)	76.54^*^	3.39	22.55	−0.23	0.21	−1.08	−32.12	28.38	−1.13	1.82	1.70	1.07

^*^Significant result *p* < 0.05. ^**^States capture racial and ethnic information in different formats. NH Other Race includes multiracial, NH Native American, and “other” categories. Removed transition month (i.e., first month of implemented increased CVB). CVB: cash value benefit; NH Non-Hispanic; SE, standard error.

#### 3.2.2 CVB dollars issued and redeemed

**Figure 3:**
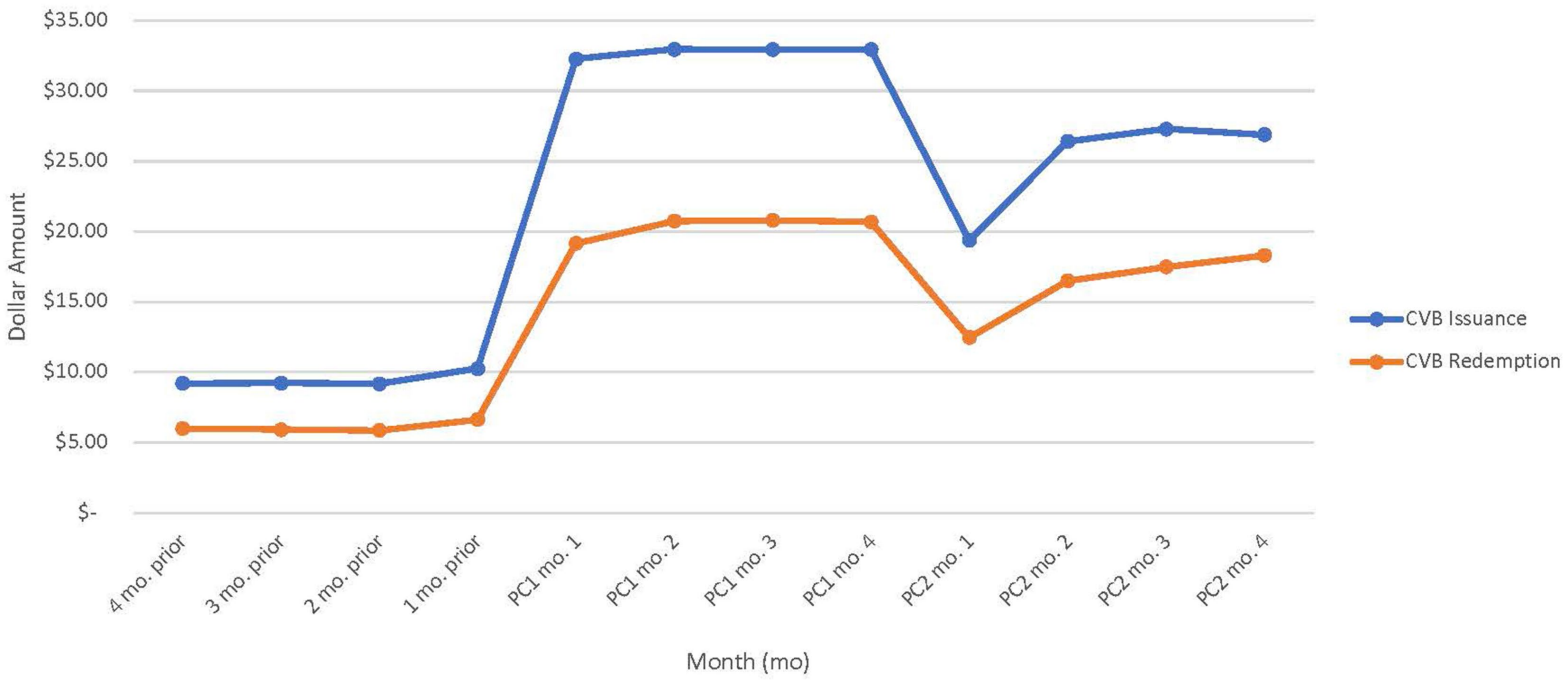
Average CVB amount issued and redeemed per WIC participant pre-implementation, during policy change (PC) 1, and PC2 (n = 23 WIC State agency datasets.) State agencies that implemented PC1 in May were excluded.

Figure 3 showcases issuance and redemption per participant for the 4 months before the $35/child/month, four months of the optional $35/child/month increase, and the first 4 months of the mandatory $24/child/month. During the change to $35/child/month, there was a stable increase in issuance (average $32.78/participant issued) and redemption (average $20.31/participant redeemed) amounts, but both sharply decreased in the first month of the $24/child/month ($19.38/participant issued vs. $12.46/participant redeemed). The issuance and redemption increased and stabilized over the next 3 months of the $24/child/month policy, as illustrated.

Overall, there was a positive association between CVB issuance and redemption. The generalized linear regression model demonstrated an increase in CVB issuance is significantly associated with higher CVB redemption (*p* < 0.001; Table 8). For every additional $1 increase in issuance, participants redeemed an additional $0.61 after controlling for number of child and adult participants, month and year, and State.

**Table 8:** Crude and adjusted generalized linear regression coefficients of CVB redemption and issuance; April 2020 to September 2022 (*n* = 810 monthly observations; 30 months times the 27 responding WIC State agencies).

Model	CVB Redemption			
	Crude β (95% CI)	*p*-value	Adjusted β (95% CI)	*p*-value
CVB Issuance	0.64 (0.63–0.65)	<0.0001	0.61 (0.60–0.63)	<0.0001

Adjusted for count of adult participants, child participants, indicator for month and year, and State indicators. Robust standard errors were clustered at the State level. CVB: cash value benefit.

### 3.3 Agency interviews

Themes from the WIC State and local agency interviews related to CVB barriers and facilitators are discussed below. The i-PARIHS subconstructs and relevant quotes are included as applicable with additional details available in Supplementary Tables S2–S5, S10.

#### 3.3.1 Implementation barriers

WIC State and local agencies faced various challenges with implementing the CVB changes. Below, we present these challenges in alignment with the i-PARIHS subconstructs of clarity; complexity; structures and systems; and time, resources, and support. Implementation barriers specific to the sub-analysis for local agencies with high participant racial diversity are also discussed.

##### 3.3.1.1 Clarity and complexity

The main challenges to implementation centered around the i-PARIHS subconstructs of clarity (i.e., degree to which implementation is understood by implementation team) and complexity (i.e., ways in which the process of implementing CVB changes is simple or complicated). Both WIC State and local agencies indicated the uncertainty of the exact changes to CVB amounts and the timing of those changes, which created difficulties for CVB implementation. With limited time to correct the benefit amount that was already programmed, some State and local agency staff had to manually reissue the correct amount. This created an excess burden on WIC staff to reissue the correct benefit amount to participants.

##### 3.3.1.2 Structures and systems

Inflexible structures and systems impacted WIC agencies’ ability to implement the CVB changes. For example, limited capability to modify the WIC Management Information System (MIS) presented barriers to implementation. WIC MIS “determines eligibility of the applicant, captures demographic data, creates, assigns, and maintains benefit prescriptions, and issues and maintains WIC cards for the WIC participant and/or WIC household” (28). State agency staff informed local agency staff of the changes to the CVB and relied heavily on local agency staff to manually reissue the benefits to reflect the correct CVB amount. One WIC State agency director indicated that “There were several [instances] where we did not know until later whether it was going to continue or not. That meant we either had to make a decision to not issue any benefits in advance or we had to issue them and void them all. That created a lot of work for either us or our local agencies because we did choose to not issue benefits in advance and issue them later, but that meant a lot of going back into clients’ records and issuing benefits manually.”

##### 3.3.1.3 Time, resources, and support

WIC State and local agencies also experienced challenges related to the i-PARIHS subconstruct of time, resources, and support (i.e., presence or absence of sufficient time/resources/support). State agencies felt their ability to successfully implement the CVB changes was often influenced by having enough staff, financial resources, and time to dedicate to implementing the CVB changes. Similarly, local agency staff that made manual updates to food packages noted difficulty finding time to do so in conjunction with other responsibilities and priorities. For example, one local agency director noted, “I would say it was a lot of work, a lot of manual work, that for us, especially in the beginning, it did take away a lot of staff time. So, it took away from doing appointments or being in our call center to do follow-up education for benefits. So, it was time intensive.” Agencies also frequently cited events, such as the COVID-19 pandemic and the infant formula recall, leading to shortages among both WIC State and local agency staff to assist with the CVB changes. Furthermore, larger local agencies were better equipped to allocate sufficient staff members to handle the manual issuance of benefits. In contrast, smaller agencies or those grappling with staffing shortages faced challenges as the burden often fell on a few staff members. This reallocation of staff time and clinic duties, including reduced availability for appointments, highlighted the need for more equitable distribution of resources and staffing support to ensure the successful implementation of CVB changes across all agencies.

##### 3.3.1.4 Implementation barriers for local agencies with high racial diversity

In a sub-analysis of agencies characterized by caseloads with high racial diversity (*n* = 30), several barriers were identified in the implementation of CVB changes. Excessive implementation workload posed a significant challenge, particularly for agencies serving a higher caseload. Caregivers, in some cases, did not receive the correct benefit amounts, leading to the need for local agencies to physically bring participants into the clinic for corrections. This process overwhelmed staff, who often lacked the necessary time, staff allocation, or resources to issue the updated food packages efficiently. Staff members expressed the need for a more streamlined implementation process to reduce these challenges. Difficulties with promotion and marketing of CVB changes were also evident. The responsibility for ensuring the correct benefit amounts were allocated often fell on WIC participants themselves, who were encouraged to contact WIC staff for clarification. This led to an increase in participant inquiries, including questions about the CVB increases. Some participants faced obstacles in receiving updates due to issues such as lacking a phone or having an unreliable phone number, resulting in missed communication efforts.

#### 3.3.2 Implementation facilitators

WIC agencies discussed several facilitators that helped them navigate the various CVB changes with key facilitators related to the i-PARIHS constructs of structures and systems; leadership support; providing education or information; and time, resources, and support.

##### 3.3.2.1 Structures and systems

WIC State agencies took a systems approach and engaged with various divisions to create resources and ensure there was capacity to implement the CVB changes. At the State level, most staff mentioned the CVB changes being an “all hands-on deck” effort by engaging all departments within their WIC unit to successfully implement the CVB changes. Examples provided included collaborations across vendor departments, MIS units to ensure changes could occur, nutrition coordinators issuing food package changes, and communication specialists to develop protocols and materials to communicate with local agency staff and WIC participants.

##### 3.3.2.2 Leadership support

WIC State and local agencies valued leadership guidance and their administrative/technical support during the CVB changes. While State staff noted frustrations with delays in receiving updates on the CVB, overall, staff found the frequent communications received from federal agency staff as supportive throughout CVB implementation. Staff specifically mentioned memos, emails, and calls as the main mode of communication provided by federal leadership. Additionally, State agency staff appreciated the willingness and availability of federal leadership to answer any questions that arose during the CVB changes. One WIC State agency highlighted that “We’re really fortunate in the [REGION] to have a very responsive office to support us. It was uncharted territory. We were all learning together, so they made themselves available.” For local agencies, the State agency provided key instructions, resources, and support for local agency staff to implement the CVB. Developing a pathway for benefits to be automatically updated in their MIS was noted as the most helpful action of the State agency, overall reducing the burden on local agency staff. In addition, State agencies providing transparent, timely communication about the CVB changes, clear directions, and being available to troubleshoot were other helpful factors for local agencies.

##### 3.3.2.3 Providing education and information

A critical component of CVB implementation was disseminating the CVB updates to WIC participants (an i-PARIHS facilitation activity subconstruct). Both State and local agencies reported playing a role in communicating these changes to WIC participants. Staff mentioned relying on various modes of communication to ensure the messaging about the changes to the CVB were reaching WIC participants. Communication channels included social media channels (e.g., Instagram and Facebook), WIC agency websites, WIC mobile apps, newsletters mailed to participants, text notifications when a benefit change occurred, and educating participants about the increased amounts during appointments. Printed and in-person promotion methods were used more often at rural agencies, and larger agencies with higher staff capacity opted to create social media graphics, recipe guides, and other resources to provide more information on how to use the increased amount. For example, one WIC State agency noted, “And that [our mobile app] is a huge, huge benefit for our participants because they can get…push notifications about this increase. When those levels changed, we had another push notification saying, ‘Hey, now it’s changed to a little bit less amount of money.’ And they can always check their benefits. Again, it’s current and up to date as of the moment you check, so you could see exactly dollar amounts that were left for the fruit and vegetable benefit.”

##### 3.3.2.4 Ideal lead time

Lastly, WIC State and local agencies were asked what the ideal lead time would be to successfully implement any future CVB changes. The most frequent response was 3–4 months with a range from one to 6 months. MIS capabilities to automatically update the CVB issuances versus manual updates was a key factor driving the amount of lead time needed. For instance, one local agency director stated, “I mean in an ideal world, we would know 3 months ahead of time, since we issue 3 months of benefits because then you do not have to redo work, or you only have to follow up on things that you might have missed.”

### 3.4 WIC caregiver interviews

Themes from the caregiver of WIC participants interviews centered around their awareness, satisfaction, and utilization of the CVB changes with relevant quotes included to support the findings. Supplementary Figure S1 provides a conceptual model for the various dynamic relationships of the WIC community across the qualitative findings. We also include findings from our sub-analysis of caregivers of WIC participants that identify as Black, Indigenous, and People of Color (BIPOC). Please see Supplementary Tables S6–S9 for additional details.

#### 3.4.1 Awareness

Similar to feedback received from State and local agencies, most caregivers reported receiving information about the CVB through WIC appointments. Caregivers in States that had the ability to send out mass texting or social media blasts noted learning of the change ahead of time through those communication efforts. One resource notably mentioned by caregivers was the WICShopper App or any application run by the State agency to help identify which fruits and vegetables could be purchased in-store. However, some caregivers were unaware that the CVB had changed and faced challenges during store checkout. For instance, one participant found out about the mandatory change to $24/child/month when they attempted to utilize the previously higher $35/child/month CVB amount issued at the store, as illustrated by the following quote: “I do not think it was until I went to go shop and hopped on the app…so it was a little disheartening because I realized I cannot get the normal stuff we would like. I could not get, if we wanted bell peppers, I probably could not have got that, just because even though it was a small cut, it still made a difference to us with the prices of everything. It’s expensive, it’s so expensive right now.”

#### 3.4.2 Satisfaction and utilization

During the interviews, caregivers were asked their level of satisfaction on a scale from 0–10 at the different CVB amounts ($9, $35, and $24). Satisfaction was the highest at 9.8 when the CVB was $35/child/month followed by a score of 7.8 at $24/child/month and then 5.3 at $9/child/month. For the $35/child/month, many caregivers reported using the full CVB amount each month and noted that the CVB amount lasted longer so they could purchase fruits and vegetables throughout the month. Caregivers reported less stress and concerns regarding affording food for their families at the $35/child/month. At $35/child/month, one caregiver noted, “So yeah, I’d get kind of the same fruit, but then I was able to afford a bag of oranges, and that makes a huge difference to my kids. And then I used a good portion of it for vegetables, and that was huge, too, because it just helped us eat correctly, even more so than before. I make sure I include mostly veggies with every meal now, and it’s a huge difference.” Caregivers were still grateful for receiving the $24/child/month CVB but noted being more selective in the fruits and vegetables they were purchasing compared to the $35/child/month CVB. Caregivers noted the $9/child/month CVB amount was used within the first two shopping trips of the month. In addition, with the $9 CVB, caregivers gravitated towards a limited selection of fruits and vegetables. If the CVB returned to the $9/child/month, caregivers noted that rising food costs and a higher cost of living would not allow the $9 CVB to stretch as far, and it would be difficult to continue offering their children fruits and vegetables in the same capacity. Most caregivers reported they would still participate in WIC if the CVB were to decrease back to $9/child/month but noted their level of utilization and satisfaction with the program would likely decrease. Though most caregivers felt the other benefits and services WIC provides are helpful, the CVB was highlighted as a key reason for participating in the program, with one caregiver stating, “The biggest reason I’m probably still on WIC is because of the fruit and veggies. I started getting on there for the formula, but this time around, I’m a breastfeeding mom…Well, I do not need formula, so why even mess with this WIC shopping? But the fruits and veggies, yeah, that’s worth staying on WIC and being on WIC, really.”

#### 3.4.3 Barriers to CVB redemption among caregivers who identify as BIPOC

A sub-analysis of interviews given by BIPOC self-identified caregivers (*n* = 46) illustrated additional barriers to using the CVB amount and supporting their families’ nutritional needs. These barriers include limited food access, lack of awareness about CVB changes, limited access to WIC-authorized vendors, and increased food expenses. Limited food access was a common challenge, as participants from Puerto Rico cited the impacts of hurricanes, which restricted the availability of fresh produce in stores, especially when the CVB amount was $9. Lack of awareness about CVB changes was another prevalent issue, with participants in Puerto Rico and some Native American participants reporting their lack of awareness about the increase to $35 and needing to contact their WIC office for information. Participants in rural areas of various ethnicities were often unaware of changes in the CVB amount and had to rely on sources like their State app, grocery store checkout, or social media for information. Limited access to WIC-authorized vendors was observed in certain rural areas, particularly among Native American participants who had access to only a few nearby stores with inconsistent stock. For example, one Native American participant noted, “There’s the closest big grocery store like…15, 20 min drive away, and there’s a little market here in town, but they almost never have anything besides prepackaged salad mixes. So fresh fruits and vegetables were something that were just…not practical for us. We needed things that would travel well and would keep well, because we could not afford to go to the store every week, especially with gas prices what they are.” Finally, increased food expenses posed a challenge in Puerto Rico, where the high cost of produce often forced participants to supplement CVB with their own funds to cover the additional expenses.

## 4 Discussion

Overall, the findings show that the implementation of the CVB policy changes at the State and local agency level had cascading effects on WIC participant CVB redemptions. A key outcome of the study assessed whether greater CVB dollar amounts increased WIC participant access to FVs, measured by FV redemption rates. The combined interpretation of the quantitative and qualitative data strands showed that the initial redemption rate decrease was likely due to the implementation challenges WIC agencies faced. Technology capabilities that allowed automatic updates to MIS were a key factor in ensuring equitable and timely issuance of the updated CVB amounts. State agencies could consider ways to leverage the lessons learned during these fast-moving policy changes to reconfigure or update MIS systems in ways that allow for increased flexibility or rapid cycle changes to CVB that are less burdensome for agencies to ensure CVD updates are available to WIC participants as soon as possible. While major changes to MIS require substantial time and financial resources, the efficiencies created over time could justify the investment. The USDA FNS WIC Modernization efforts and related funding could be resources for WIC State agencies when upgrading their current MIS (29).

Communication with WIC participants also was a critical factor in the success of CVB implementation with some agencies reporting delays in communicating the changes to WIC participants. Technology-based communications, such as social media and WIC-specific apps, could help with increasing the efficiency and effectiveness of communications with WIC participants. These types of communications were reported as positive by both the WIC agencies with these capabilities and their WIC participants. This finding is supported by other research that shows high WIC participant utilization and satisfaction with WIC apps (30, 31). However, the current study observed inequities across agencies regarding their ability to use the functionalities of WIC-specific apps to communicate with their participants. Nevertheless, there are some cases where printed communications are beneficial due to issues with internet access and how the specific WIC community typically receives information. For example, some ITO agencies indicated a preference for using local newspapers and radio stations to reach WIC participants that reside on tribal lands.

Yet, the quantitative results found that redemption rates slightly increased after the initial transition period of implementing the CVB changes. During the WIC State and local agency interviews, respondents indicated that more WIC participants redeemed higher dollar amounts of the CVB after the change to $35/child/month. The WIC participant interviews also demonstrated that caregivers of WIC participants utilized the increased CVB amounts with many noting that they recalled redeeming nearly the entire $35/child/month or $24/child/month.

Furthermore, WIC participants showed a strong preference for the CVB of $35/child/month to purchase a sufficient amount and variety of FVs to yield a meaningful impact on their families’ diet. These findings align with the previous studies that showed the $35/child/month increased access and redemption of FVs along with an increase in WIC participant satisfaction (14, 16, 32–34). While the caregiver interviews were overwhelmingly positive, some caregivers faced external, structural barriers to CVB redemptions (e.g., lack of awareness of the CVB changes, lack of access to WIC retailers, and inadequate amounts of FVs at WIC retailers). A recent study in North Carolina reported similar barriers with WIC participants experiencing difficulties finding sufficient FVs to fulfill the higher CVB amounts (15). These findings stress the importance of WIC agencies and retailers working together to ensure the proximity of WIC-authorized stores to their WIC participants and that those stores are stocked with enough FVs. A best practice reported by some WIC agencies in the current study was consistent communication with WIC-authorized retailers so that the retailers could adjust their stock of FVs.

Disparities were observed when analyzing both the quantitative and qualitative data by race and ethnicity. Constrained resources and staff capacity among WIC agencies with more ethnically diverse caseloads and structural barriers to redeeming the full CVB benefit among caregivers of WIC participants who identified as BIPOC could help explain some of the differences in redemptions observed in the quantitative data. Disparities in redemption rates existed prior to the COVID-19 CVB changes with a 2015 study of Virginia WIC participants reporting that the lowest FV redemption rates were among African American and Black participants (35). Implementation of policy changes, such as the changes to the CVB amounts, should incorporate equity-focused strategies (35, 36) to help resolve the barriers to and inequities of CVB redemptions.

The study includes limitations to consider when interpreting the findings. First, the results are not based on a nationally representative sample with purposive and convenience sampling used for the interviews with WIC local agencies and participants. While the research team implemented strategic efforts to collect responses from a diverse sample of WIC State agencies, local agencies, and caregivers of WIC participants, the findings do not represent all WIC agencies and participants nationwide and could be driven by the experiences of respondents that agreed to participate in the interviews. Furthermore, other factors during the data collection period could have impacted the findings, such as the infant formula recall, remote WIC services, and COVID-19-driven food cost inflation. Despite the limitations, the study included several strengths. Incorporating feedback from an advisory panel comprised of WIC State agencies, local agencies, and WIC participants helped ensure that the data collection instruments and protocols and study findings were tailored and applicable for the intended audiences. An innovative aspect of the study was the use of use the I + PSE framework which proved to be well-suited to address the study objectives and goals due to its ability to use systems thinking to assess the multidimensional impacts of CVB policy changes. This framework enabled the comprehensive identification and analysis of multiple factors influencing CVB uptake at the WIC State agency, local agency, and participant levels.

This research aimed to examine CVB implementation from the perspective of the individual, practice, program, organization, policy, and population levels per the I + PSE framework for Action, but retailer perspectives and impacts were beyond the scope of this project. Future research should aim to examine the national economic contribution of changes to the CVB for WIC-authorized retailers. Retailers are an integral part of the WIC program. Still, the program has inconsistent requirements across states and a high regulatory burden for retailers (37), which may deter retailers from accepting WIC benefits as payment. Therefore, future exploration of the impact of the CVB from their perspective could strengthen the argument for retailers to consider gaining or maintaining the WIC-authorized status.

## 5 Conclusion

The COVID-19 pandemic prompted a series of quick-turnaround policy changes across federal nutrition assistance programs, including WIC. This is one of the first studies to our knowledge that sought to understand if the policy changes to the WIC CVB for fruits and vegetables were implemented as intended at the WIC State and local agency level. Policy makers have a unique opportunity to help increase access to healthy food for millions of families nationwide. Despite some implementation challenges, the increased CVB, especially at $35/month, shows promise in improving WIC participants’ fruit and vegetable access and overall satisfaction with WIC. In addition, policy makers and federal agencies could coordinate efforts to ensure that WIC agencies receive adequate lead time for implementation of food package changes to help minimize errors or disruptions in the issuance of benefits. Also, WIC agencies with limited financial and technical resources need additional support, including funding for staff training, system upgrades, and the creation of educational materials for participants, to help ensure equitable distribution and utilization of the FV benefits. Lastly, researchers can continue to conduct studies to identify emerging barriers and facilitators when implementing WIC program changes, especially given the upcoming WIC food package revisions. In addition, research gathering WIC retailers’ experiences with WIC food benefit changes is also critical to identify ways to increase WIC families’ access to FVs.

## Data availability statement

The datasets presented in this article are not readily available because due to data confidentiality and privacy concerns. The Supplementary Tables include the data that can be shared. Requests to access the datasets should be directed to anitto@centerfornutrition.org.

## Ethics statement

The requirement of ethical approval was waived by University of Nebraska Medical Center Office of Regulatory Affairs for the studies involving humans because it was determined that the study was program evaluation, and thus, did not meet the definition of research that requires IRB review and approval, as defined in the regulations at 45 CFR 46.102(l) and OHRP Guidance. The studies were conducted in accordance with the local legislation and institutional requirements. Written informed consent for participation was not required from the participants or the participants' legal guardians/next of kin because respondents were provided information about the interview at the beginning and gave consent by agreeing to proceed with the remote interviews conducted over telephone or Zoom.

## Author contributions

AN: Writing – original draft, Writing – review & editing. MC-B: Writing – original draft, Writing – review & editing. JY: Writing – review & editing. EA: Writing – review & editing. SK: Writing – original draft. DV: Writing – original draft. JH: Writing – review & editing.
