# Supplementary material for: Mixed methods evaluation of the COVID-19 changes to the WIC cash-value benefit for fruits and vegetables
Source: Front Public Health. 2024 Apr 29;12:1371697. doi: 10.3389/fpubh.2024.1371697 (PMC11089207; doi:10.3389/fpubh.2024.1371697)
Supplement: Supplementary file 1 [file Data_Sheet_1.pdf]

Supplementary Material

1 Supplementary Figures and Tables

1.1 Supplementary Figures

1.2 Supplementary Tables

frontiers

1.1 Supplementary Figures

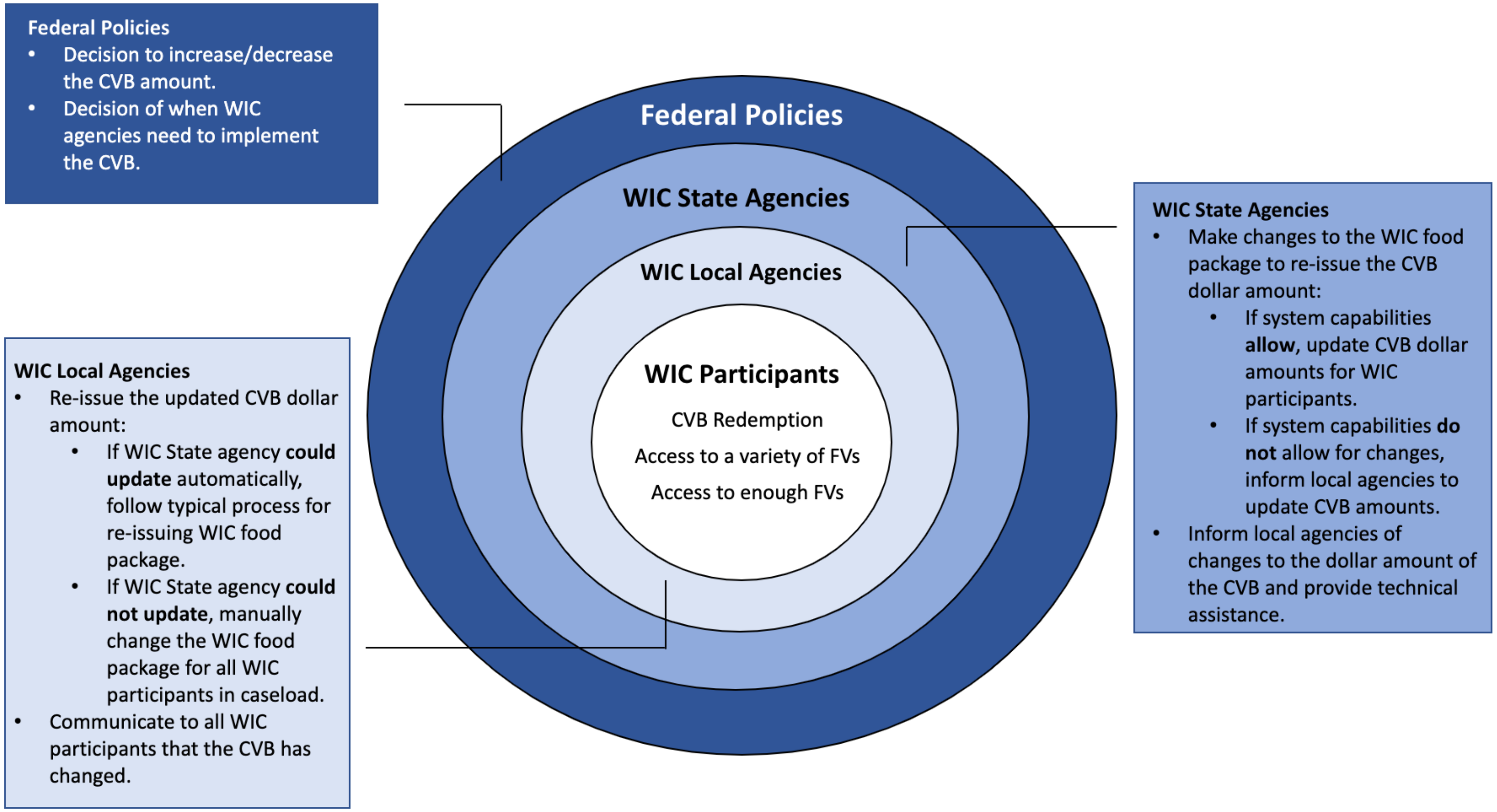

Figure 1. Multi-level Conceptual Model for Changing WIC CVB

1.2 Supplementary Tables

Table 1. Characteristics of the WIC State Agency Dataset and Overall WIC State Agencies

State Agency Characteristics	State Agency in Dataset (n=27)	Overall State Agency (n=89)
FNS Region [n (%)]		
Mid-Atlantic RO	1 (3.7)	8 (8.9)
Midwest RO	5 (18.5)	7 (7.9)
Mountain Plains RO	3 (11.1)	18 (20.2)
Northeast RO	3 (11.1)	10 (11.2)
Southeast RO	2 (7.4)	11 (12.4)
Southwest RO	5 (18.5)	24 (27.0)
Western RO	8 (29.6)	11 (12.4)
Type of Agency		
State	23 (85.2)	50 (56.2)
ITO/Territory	4 (14.8)	39 (43.8)
Caseload		
<10,000	5 (18.5)	39 (43.8)
10,000-75,000	15 (55.6)	40 (44.9)
>75,000	7 (25.9)	10 (11.2)
Opted into $35/child/month		
Yes	27 (100.0)	85 (95.5)
No	0 (0.0)	4 (4.5)
Type of data*		
Overall data only	15 (55.6)	NA
County-level	12 (44.4)	NA
Race/ethnicity	11 (40.7)	NA
FV subcategories	8 (29.6)	NA

*Not mutually exclusive. CVB: Cash value benefit; ITO: Indian Tribal Organization; RO: Regional Office

Table 2. WIC State agency and ITO/Territory staff experiences with implementing the Cash Value Benefit (CVB)

**i-PARIHS Construct**	**i-PARIHS Sub-construct**	**Themes**	**Description**	**Quotes**
Characteristics of the Innovation	Clarity<br><br>Complexity<br><br>Usability	State agencies reported needing more clarity around the timeline for implementing CVB change.	Both WIC States and ITOs/Territories experienced CVB implementation challenges caused by congressional delays creating an uncertain timeline for issuing the correct CVB amounts. As a result, State agencies were notified of the change to the CVB and given limited time to correct the benefit amount that was already programmed, causing either State or local agency staff to manually reissue the correct amount. This created excess burden on WIC staff to re-issue the correct benefit amount to participants.	"Our participants in October of 2021 did not get the higher level of benefits. It was deeply disappointing for all of us because we didn't know until it was sort of too late. We were already into October. Then, while some agencies could have had the staff capacity to go in and change benefits for families who had benefits issued yet in the month, we made a decision that we felt that created a lot of inequity, that some families were going to get the higher amount and some families weren't and there wasn't a good way that we can explain that." – State agency staff (>75K caseload)<br><br>"There were several [instances] where we didn't know until later whether it was going to continue or not. That meant we either had to make a decision to not issue any benefits in advance or we had to issue them and void them all. That created a lot of work for either us or our local agencies because we did choose to not issue benefits in advance and issue them later, but that meant a lot of going back into clients' records and issuing benefits manually." – State agency staff (<10K caseload)

	Degree of Fit<br><br>Complexity	State agencies found their ability to issue the correct CVB amount in a timely manner was highly dependent on management information systems (MIS) capabilities.	MIS were a crucial component in State agencies' ability to issue the correct CVB amount in a timely manner. In some cases, where MIS couldn't handle the CVB change automatically, State agencies had to update and re-issue the correct CVB benefit amount. As a result, State agency staff informed local agency staff of the changes to the CVB as soon as possible, and then relied heavily on local agency staff to manually reissue the benefits to reflect the correct CVB amount.	"We had probably 500 or 600 or more instances where we had to manually change their benefit level because it was issued incorrectly. With our MIS system, if any one piece of those aggregated food benefits had been redeemed, we can't just go in and increase that." – State agency staff (10K-75K caseload)<br><br>"There was a lot of workarounds that people had to do to make it all work. There's been a lot of discussions about the MIS and how the one we have is not the most nimble and agile to respond to these kinds of changes super quickly. I think that made it challenging because we couldn't just go in and click and reissue all this. It literally was very manual." – State agency staff (>75K caseload)
	Trialability<br><br>Relative Advantage	Being able to test and adapt procedures after experiencing one CVB increase facilitated implementation success for future CVB changes among State agencies.	Only 4 out of the 23 State agencies interviewed had the capacity and ability to test MIS systems prior to any CVB changes being implemented. This allowed MIS consultants to run and test various scenarios to ensure automatic implementation of the CVB would run smoothly. As a result, there were no barriers or challenges for those State agencies when issuing the CVB changes. For those who were implementing the CVB changes manually, staff noted that after experiencing one CVB change, they	"We've done this [implement the CVB changes] so many times now, I feel like we've refined a business process for how we implement things. If there was a silver lining to all of this, I think that was maybe my takeaway like we now have a methodology for when we need to quickly implement something…" – State agency staff (10K-75K caseload)<br><br>"The programmer has to spend a lot of time testing it in our test and development environments…we probably use three or four of people

			were able to reflect and internally modify procedures to ensure implementation of future CVB changes would run smoothly.	and we go through in those test environments, and we have to go through and make sure, 'Did the script run correctly? Did it do what it should do?'" – State agency staff (>75K caseload)
Characteristics of the Recipient	Time, Resources, Support	State agencies felt their ability to successfully implement the CVB changes was often influenced by having enough staff, financial resources, and time to dedicate to implementing the CVB changes.	State agencies noted financial restraints prohibited them from being able to update MIS systems to automatically issue the CVB benefits. Additionally, State agencies frequently cited events such as the COVID-19 pandemic and the infant formula recall leading to shortages among both WIC State and local agency staff. As a result, State agencies noted they could not allocate enough staff to assist with the CVB changes which hindered implementation.	"Our case load has been growing during COVID this whole time, and they've had staffing issues and people have been out sick, and then the formula thing happened. There's just been a lot of stressors on the program. I think it was very stressful to have to do all this manual work in addition to all the other work." – State agency staff (10K-75K caseload)<br><br>"We had the people we needed. What it did is it took us away from very other important other work. What it meant is we had to give stuff some stuff up because this was a mandatory implementation, we had no choice." – State agency staff (10K-75K caseload)
Systems and Structures	Infrastructure, Resources, and Support<br><br>Structures and Systems	WIC State agencies took a systems approach and engaged with various divisions to create resources and ensure there was capacity to implement the CVB changes.	At the State level, most staff mentioned the CVB changes being an "all hands-on deck" effort by engaging all departments within their WIC unit to successfully implement the CVB changes. Examples provided included: working with vendor departments, MIS units to ensure changes could occur, nutrition coordinators issuing food package changes, and communication	"The whole nutrition division was involved in some way. The sales department provided guidance to the vendors. The finance department dealt with the allocation of additional funds and ensured proper use of them. I'd say basically the whole staff was involved in some way or another." – ITO/Territory staff (>75K caseload)

			specialists to develop protocols and materials to communicate with local agency staff and WIC participants.	"So, October of 21 is when the first big CVB push at the state and at the local clinics. That was definitely an all hands on deck because we didn't really know how to implement it to be able to get everyone in a timely fashion." – State agency staff (10K-75K caseload)
	Leadership Support	Guidance and support provided by agencies at the federal level was valuable to State agencies during the CVB changes.	While staff noted frustrations with delays in informing them of changes to the CVB and when to issue the amount, overall, staff found the frequent communication they received from federal agency staff as supportive throughout the implementation of the CVB. Staff specifically mentioned memos, emails, and calls as the main mode of communication provided by leadership at the federal level. Additionally, State agency staff found the willingness and availability from federal agency leadership to answer any questions useful during the CVB increases.	"We're really fortunate in the [REGION] to have a very responsive office to support us. It was uncharted territory. We were all learning together, so they made themselves available. They provide guidance, they do not provide decisions, but they listen to what the concerns are and give some examples of how you might go about addressing that, but in a lot of ways, sometimes they didn't have any more information to share, and they weren't able to answer questions because it was unchartered. They were very open to us saying, 'Well okay, based on that, this is how we're going to approach it,' and then often shared that information with the national office." – State agency staff (10K-75K caseload)<br><br>"I think the communication that we did receive from USDA headquarters, that was very clear and concise to the point that the decisions were made. But it was, again, that time period is very challenging. States were doing things all over the place when it had came to, you're going to keep the higher level,

				you're going to the lower level, you're going to gamble, you're not going to gamble, kind of thing." – State agency staff (10K-75K caseload)
	Networks and Relationships	State agencies leveraged existing networks and relationships with other agencies to share information and facilitate implementing the CVB changes.	Leveraging partnerships outside of the agency were crucial for implementing the CVB changes, such as leveraging vendor partners and other WIC State agencies. Staff members also mentioned the social media resources and toolkits provided by the National WIC Association (NWA) were beneficial in communicating changes to local agency staff and WIC participants as well.	"The National WIC Association is a huge resource for us in WIC. They were really helpful for us as state agencies in clarifying for us what was going on at the national level policy-wise, what they were hearing from USDA. Also, they offered opportunities for us as states to kind of share approaches with each other, ask questions of each other. We have WIC director calls every other Friday and we have since the pandemic started and that's been a huge help." – State agency staff (>75K caseload)<br><br>"WIC is changing, and they're just used to that. It's just so fluid in everything that they do. And they're so remarkable at being able to come up with these solutions. If I was a standalone state agency and I didn't have the other six ITOs to work with and have that kind of continuous support, I think I'd have got lost in all of this." – ITO/Territory staff (<10K caseload)
Facilitation Activities	Providing Education or Information –	State agencies emphasized the importance of frequent	To do their best to address the uncertainty surrounding the CVB timeline, State agencies frequently provided transparent communication	"Those calls/webinars became monthly probably around fall of 2021. Our updates and discussion with our local agencies on those monthly calls has

	Educating Staff Problem Identification and Resolution	communication and resources containing information regarding the CVB changes to support and address any challenges local agencies encountered.	and educational resources on how to implement the CVB changes to local agencies. These resources typically provided information on how to manually change the CVBs to reflect the updated amount for agencies that could not automatically issue benefits. Modes of educational materials included newsletters, monthly meetings with local agency nutrition coordinators or directors, and email blasts as soon as State agencies were notified of a CVB change.	been a big vehicle for us to share information with them, allow them to ask questions, because we always leave time at the end for it to be interactive and take questions and comments. We also relied a lot on our weekly e-newsletter that we sent to local agencies. So, we would put guidance and information in there." – State agency staff (>75K caseload)<br><br>"Our nutrition and breastfeeding unit was responsible for putting together documents or guidance for our local agencies, for staff, so that they are aware. Because it's not easy to work in a local agency if you're not understanding yourself, and you're trying to explain it to participants who are the actual end user of this wonderful new benefit." – State agency staff (10K-75K caseload)

ITO: Indian Tribal Organization

Table 3. WIC State agency and ITO/Territory staff perceived impact of cash-value benefit (CVB) implementation on participants' experience, fruit and vegetable access, and CVB redemption rates

i-PARIHS Construct	i-PARIHS Sub-construct	Themes	Description	Quotes
Characteristics of the Innovation	Observable Results<br><br>Degree of Fit	As a result of the changes to the CVB, State agencies saw a higher dollar redemption of the CVB among WIC participants.	Due to the increased CVB amounts, both State agency and ITO/Territory staff reported more WIC participants were redeeming higher dollar amounts of the CVB each month. Staff heard success stories of WIC participants incorporating more fruits and vegetables into their diet and were able to expose their children to new fruits and vegetables. WIC State agency staff mentioned the CVB changes having a positive impact on participants aligned with WIC program goals to ensure individuals had access to healthy foods, as well as exposing children to a variety of foods early on in life.	"From a participant perspective, we did see pretty good redemption rates of the increased CVB. We did track it, not just by child category, but we could do that. But we did check that. And then, it was also neat to hear just the participants' feedback about, because there were more dollars, they were more willing to try new items because, even if their child didn't like, it wasn't like they were going to go without because of the increased amount. And so, we did a lot of work around ideas for spending your CVB." – ITO/Territory staff (<10K caseload)<br><br>"We got lots of communications like, 'We were able to try different vegetables because we've never had the money before.' 'My kid got cherries for the first time.' I will say just as an aside, during the CVB thing, lots of times I'll grab some fruit or something and I grabbed some cherries for them one day and it rang up at 14 bucks. And I was like, 'If the kiddos were still on $9, they might not be able to experience these cherries like we're getting to.'" – State agency staff (10K-75K caseload)
	General Attitudes	State agencies felt motivated to ensure the CVB was implemented correctly for all WIC	Generally, WIC staff were excited and emphasized the benefit the CVB increases had on providing more money for	"I know one thing we were kind of talking about, it made it really pretty easy to promote it. Because I feel like there was a lot of staff buy-in. Staff were really excited about this

		participants to access and use.	WIC participants to spend on fruits and vegetables. Increases to the WIC CVB led to increased redemption rates among WIC participants which led to staff feeling motivated to continue implementing increased benefit amounts.	change for clients, and they really promoted it." – State agency staff (10K-75K caseload)<br><br>"I have to give my staff a huge amount of credit. I mean, they never blinked an eye. They were just like, 'Yes, let's do this. This is great for family. This is great for kids.' And just their willingness to jump in was great." – ITO/Territory staff (<10K caseload)
Facilitation Activities	Providing Education or Information – Marketing to Participants	State agencies relied on various modes of communication to inform WIC participants of the CVB changes to ensure they were utilizing benefits to the fullest amount.	Disseminating information regarding the CVB increases to WIC participants was of high importance to State agency and ITO/Territory staff members. Staff mentioned relying on various modes of communication to ensure the messaging about the changes to the CVB were reaching WIC participants. Communication channels included social media channels (e.g., Instagram and Facebook), WIC agency websites, mobile apps, newsletters mailed to participants, text notifications when a benefit change occurred, and educating participants about the increased amounts during appointments.	"A lot more people [were] asking about what other foods I can try or how I could cook this fruit or this vegetable or more questions on how to eat it or what I can mix it with and all this stuff. So that was interesting. Usually, we kind of try to sneak that into our newsletter, just kind of let everybody know about it, but we had people asking us so many questions that we were actually just taking from what they were asking us, and we were telling them and putting it in our newsletters and sharing more recipes more than monthly." – ITO/Territory staff (<10K caseload)<br><br>"And another piece that we haven't mentioned yet is our mobile app. And that is a huge, huge benefit for our participants because they can get, we did have push notifications about this increase. When those levels changed, we had another push notification saying, 'Hey, now it's changed to a little bit less of fund amount of money.' And they can always check their benefits. Again, it's current and up to date as of the moment you check, so you could see exactly dollar amounts that were left for the fruit and

				vegetable benefit." – State agency staff (10K-75K caseload)

ITO: Indian Tribal Organization

Table 4. Local agency staff experiences with implementing the Cash Value Benefit (CVB)

i-PARIHS Construct	i-PARIHS Sub-construct	Themes	Description	Quotes
Characteristics of the Innovation	Degree of Fit<br><br>Usability	Local agency staff noted differences in technology needs, time required, and difficulty to implement the higher CVB amounts compared to the process for the $9 CVB amount.	Implementing the new CVB amounts often required new processes for local agencies, particularly for those that had to make manual updates in their management information systems (MIS) to issue the new amounts. Time was a frequently mentioned barrier to implementing the CVB since updating the food packages was done in addition to their typical workload. System delays, varying benefit amounts, and difficulty adopting a new procedure were other challenges faced by local agency staff. Clinics that had their benefits automatically updated and issued noted fewer challenges with implementation.	"Oh my gosh, it was awful [making those manual changes]. It slowed everything down. Benefits couldn't be issued without a CPA, so before the process and assistant or the clerk could just issue benefits, but everybody had to go through a CPA so that she or he could manipulate the food package and increase it. So, it definitely bottle necked clinic flow." – Nutritionist (mostly rural, 750-1,999 caseload)<br><br>"I hate to sound so simple, but I feel like it was one of those cases where once we got the different steps and instructions about how much and when those would be implemented, it was just kind of like, okay, we're just going to kind of do it. So again, I had a couple of site supervisors who were the lead in making sure that staff had all the information and tools that they needed. But for us it was a very simple process." – Local agency director (mostly urban, 2,000-4,499 caseload)
	Clarity<br><br>Complexity	Local agency staff mentioned confusion with the implementation process due to frequent policy changes that led to frequent procedure changes.	Delayed notice of policy changes and frequent policy changes related to the CVB led to uncertainty and confusion among local agency staff about the correct timing and benefit amounts. Due to this, staff noted difficulty planning ahead and staying updated on the	"One thing that was just really difficult overall was the fact that we... I think this tends to happen, but we didn't find out what was happening until a month or so before or a couple weeks before, and so it was really hard to plan when we weren't sure if it was going to continue, if the legislature was going to pass to keep it on, how long was it going for?" – Local

			correct implementation procedures.	agency director (mostly urban, 2,000-4,499 caseload)<br><br>"So on top of CVB, we also have changes in other pieces of the package that have then lend it to be like, 'Oh, package Y is what you need to choose, but it's now package Y-ABC.' So it's a lot on CPA staff....So it does take a lot of extra work. And there was a time, and currently we're in this time again, where within a three-month span of issuing packages, all three months have a different package. Because one month might be $24, you can see. And then at some point it switched to $25." – Local agency director (mostly urban, 2,000-4,499 caseload)
Agency Staff	Skills and Knowledge<br><br>Collaboration and Teamwork	Implementing the CVB changes required collaboration between multiple levels of local agency staff and various strategies to ensure effective and timely execution.	All levels of staff were typically involved in the CVB changes with staff in leadership roles completing coordination and management tasks, dietitians and competent professional authorities (CPA) providing direct client care and issuing benefits, and administrative staff facilitating communication. Larger agencies were more likely to maintain distinct roles throughout the CVB changes, while smaller agencies with limited staff capacity often	"So, my role was mainly ensuring that the participants did get the benefits that they should be receiving with that increase, and so what that looked like for us was receiving a document from the state office, and then I assigned it out to staff members and/or helped myself to actually make the adjustments within our system." – Local agency director (mostly urban, 2,000-4,499 caseload)<br><br>"So currently, I am [COUNTY] WIC coordinator for our WIC office. So in the recent months, I've actually also been the actual CPA, providing services to our clients. We are actually in the process of hiring two new staff people. So I've kind

			adopted an all-in approach to implement the CVB.	of been doing pretty much everything in the clinic." – Local agency director (mostly urban, less than 750 caseload, ITO/Territory)
	Time, Resources, Support	Local agency staff that made manual updates to food packages noted difficulty finding time to do so in conjunction with other responsibilities and priorities.	Receiving short notice to implement the new CVB amounts created additional challenges for local agency staff that manually updated the food packages, and some noted that this task took away time from appointments, reduced or removed their typical down time, and required additional work days. Staff capacity and staffing changes at the agency also influenced whether deadlines could be met.	"I mean, I would say it was a lot of work, a lot of manual work, that for us, especially in the beginning, it did take away a lot of staff time. So, it took away from doing appointments or being in our call center to do follow-up education for benefits. So, it was time intensive." – Local agency director (mostly urban, more than 4,500 caseload)<br><br>"So, I think it was a hard discussion and it was, I would say, a little bit uncomfortable too, because I was coming from a large agency where staffing was a concern. We were short-staffed, yet we were also having to pull to do all these corrections. And so, it was really hurting us quite a bit." – Local agency director (mostly urban, more than 4,500 caseload)
Context	Leadership Support	Support from the State agency played a critical role in making implementation a success for most local agencies.	Guidance and communication from the State agency provided key instructions, resources, and support for local agency staff to implement the CVB. Developing a pathway for benefits to be automatically updated in their MIS was noted	"They definitely were communicating with us via email to inform us... This way they could give the information to us and their marketing team would also share information and signs and graphics for us to share with families. They were always quick to inform us of any changes that were final. Then, because of course, when

			as the most helpful action of the State agency, overall reducing the burden on local agency staff. Providing transparent, timely communication about the CVB changes, clear directions, and being available to troubleshoot were other helpful factors.	things change the government, it still takes time for the developers to input it in the system, so they were updating us as to when it would be official for us to be able to give to our families. Yeah, the state was great in just informing us." – Nutritionist (mostly urban, more than 4,500 caseload)<br><br>"Well, if there were going to be a change, it already be a procedure. We in the clinic can't handle it anymore. So that would be done remotely by those who support us in the system so that they can migrate that information to the EBT card. Because we can't handle that change in value here, it has to come from the state agency, they would have to handle that." – Other key staff (mostly rural, 750-1,999 caseload, ITO/Territory)
	Organizational Priorities	Managing other priorities, such as navigating the pandemic, the formula shortage, and other changes to WIC services, became a barrier to CVB implementation for some local agencies.	Local agencies with registered nurses on staff were often pulled from the clinic to assist with COVID-related tasks, taking away staff capacity in the WIC clinic to implement the CVB. Similarly, the formula shortage led local agency staff to take some attention away from the CVB to help participants find what they needed. Some clinics also noted changes to their MIS, communication channels, and updates to other services that	"Right away, during the increase, we were kind of being pulled in other directions 'cause we are a public health and our WIC agency employees nurses, so we were being pulled in to help with [COVID] vaccines and testing and things like that." – Local agency director (mostly rural, 750-1,999 caseload)<br><br>"We had the formula thing. Yeah, recall we are a Similac state and I know that every state fell into it. It didn't take long for all the other companies to not have enough either. So yeah, I think keeping our head above water with CVB while

			they had to manage in addition to the CVB changes.	answering the phone calls about the formula crisis when your attention is [split].” – Local agency director (mostly urban, more than 4,500 caseload)
Facilitation	Problem Identification and Resolution	Local agency staff developed specific procedures for issuing benefits and informing participants of CVB changes to minimize the risk of participants not being able to use their full benefit amount.	Frequent policy changes and delayed communication about the changes led to some participants not receiving the higher benefit amounts or receiving an incorrect amount. To mitigate this risk, local agency staff used methods to check benefit amounts more often such as creating system reminders, running reports, and limiting how far in advance they issued the higher benefit amount. Agencies that primarily relied on in-person communication prior to the pandemic began to use phone calls, mail, and other digital communication channels to inform participants of the changes.	“We had a lot of questions when we could not preload for three months all the higher numbers, then we would have people asking us constantly, ‘I think you messed up on my benefits. I only have $9.’ So we answered a lot of those questions, like the staff would have to. And that we would say, ‘Well, we’re hoping it will change. Stay tuned.’ And so that was a difference, I would say, for us.” – Local agency director (mostly urban, more than 4,500 caseload)<br><br>“Right away, during the increase, we were kind of being pulled in other directions ’cause we are a public health and our WIC agency employees nurses, so we were being pulled in to help with [COVID] vaccines and testing and things like that.” – Local agency director (mostly rural, 750-1,999 caseload)

	Providing Education or Information – Educating Staff	Regular communication among all local agency staff about CVB implementation procedures and updates provided staff with necessary information and training.	Scheduled staff meetings and frequent email updates sent by the local agency directors served as training for all staff to stay informed on the CVB changes and provided an opportunity to ask questions. This helped staff become more familiar with the procedures to update benefit amounts and notify participants, and training became less frequent as time went on.	"Yeah, the first bump was a little bit more of a training, how it would appear on their benefits, what's the timing on the additional amount, what kind of communication are we going to provide to folks? But then after the first bump or the first extension, it became more of communication via email." – Local agency director (mostly rural, 2,000-4,499 caseload)<br><br>"So the staff training was a lot of, please go back through emails, through staff meetings, through every contact we had. I know we're working hard and we appreciate it. Keep paying attention, keep paying attention, keep looking at the CVB amounts, keep looking. Is it the right amount? Have you issued the right amount?" – Local agency director (mostly urban, more than 4,500 caseload)

Table 5. Local agency staff's perceived impact of cash-value benefit (CVB) implementation on participants' experience, fruit and vegetable access, and CVB redemption rates

i-PARIHS Construct	i-PARIHS Sub-construct	Themes	Description	Quotes
Characteristics of the Innovation	Observable Results	Despite some implementation challenges, many local agency staff noted the increased CVB led to increased or consistent rates in participation and redemption and increased overall satisfaction among participants.	With the higher CVB amounts, local agency staff noted that participants expressed their gratitude and excitement to have access to more fruits and vegetables compared to the $9 CVB amount. This contributed to more participants being active in the program and utilizing their benefits more often in most cases; however, some agencies noted their rates remained consistent pre- and post-CVB changes. Challenges with implementation such as confusion or lack of awareness among participants about the changes and difficulty issuing benefits could have been an influencing factor.	"I feel like CVB values or CVB benefits have always been one of the first things to be redeemed on participant packages. So with the higher value, I feel like it's going even faster. It's also been one of the main reasons that we have families who will... They may have not been on WIC for a while and just kind of felt like, 'Yeah, it's not really worth it.' We've had a lot of them come back and that's been really helpful for participation as well." – Nutritionist (mostly urban, more than 4,500 caseload)<br><br>"I think that our families used those dollars and that it definitely impacted, and some people probably stayed on WIC longer because of the increased dollar amount, made it worth it to them to continue to get WIC where sometimes they'll drop off if they feel like what they're getting isn't really worth the time and effort that it takes to get WIC." – Local agency director (mostly urban, more than 4,500 caseload)

	Degree of Fit	Participants reported to local agency staff that they were more likely to reach their nutrition goals around fruits and vegetables with the higher CVB amounts.	Local agency staff noted that they often set nutrition goals with participants related to increasing fruit and vegetable consumption and the higher CVB helped many of these goals to be achieved. This also helped to solidify nutrition education and removed the access barrier so participants could apply their new knowledge.	"I would say just seeing the goal setting aspect of things that more clients are just, their goal is to eat more fruits and vegetables and just for them to have the ability to meet that goal through the increase in the dollar amount for the CVB, I think that's just really, a plot positive." – Nutritionist (mostly urban, more than 4,500 caseload)<br><br>"It was very interesting to see how much more of specific types of fruits like berries or the more expensive fruits that were being redeemed versus just getting bananas and things like that. So I feel like it was really used and utilized more. I mean, very frequently. I'm not sure what it was like previously because it was before me, but it was utilized a lot in our participation rate, is very high for that." – Nutritionist (mostly urban, more than 4,500 caseload)

Agency Staff	General Attitude	Local agency staff largely had positive feelings about the CVB increases and felt this was a beneficial change for participants.	Local agency staff mentioned several benefits of the higher CVB for participants including increased buying power to minimize impacts of rising food costs, increased value of the food package and the WIC program overall, and better alignment with a nutrient rich diet. Some noted that decreasing the CVB back to $9 would have an impact on their health and likely decrease the appeal of the program.	"So for me, it is really important that they see the value on the nutrition counseling and the food that they're getting to understand that the access to the food that they're getting, it's so important for their health, the children's health, because they have access to healthy food no other program can offer." – Local agency director (completely rural, less than 750 caseload)<br><br>"[If the CVB were to decrease] And so, I think we would lose a lot of families that came on. And I think it's just more reflective of what we should be providing. Kind of sad that we provide a lot of juice and a lot of milk, but not a lot of fruits and vegetables." – Local agency director (mostly urban, more than 4,500 caseload)
Context	Infrastructure, Resources, and Support<br><br>Political Factors and Dynamics	State WIC policies and limited access to resources prevented some local agencies from promoting the CVB changes on a large scale (i.e., mass text messaging, social media), creating challenges for participants to be informed of and redeem the CVB.	Some local agencies did not have control over their promotion methods due to state-specific WIC communication policies and had to solely rely on their State agency to promote the CVB. This created challenges for local agency staff when there were delays in when the information was shared by the State agencies and participants would call the clinic in large volume with	"Just because we're still in the process of trying to get text messages approved, and there's always some red tape when it comes to what we can put on social media, I think that could... Because if we had text messages approved by that time, then we could have just sent this big blanket text message out. Because we're delayed in social media, we're just not up to par

			questions. Clinics that did not have access to a mass communication system noted that it was difficult to inform all participants of the changes since staff did not always have the capacity to reach out to participants individually.	with some other states and their communication tools and that affected that as well." – Nutritionist (mostly urban, more than 4,500 caseload)<br><br>"We do have text communication in forms of appointment reminders and we have our Facebook page. But again, like if we wanted to get Instagram, there are so many approvals and things like that, that we have to go through in order to do that." – Nutritionist (mostly urban, 2,000-4,499 caseload)
Facilitation	Providing Education or Information – Marketing to Participants	A variety of methods were used by local agencies to inform participants of the CVB changes and encourage use of the increased amount.	The primary method for informing participants about the CVB changes was through appointments alongside nutrition education around fruits and vegetables. Social media, emails, and mailed flyers were also widely used by local agencies, and a smaller number of clinics utilized mass text messaging. Printed and in-person promotion methods were used more often at rural agencies, and larger agencies with higher staff capacity opted to create social media graphics, recipe guides, and other resources to provide	"[The CPAs] were issuing and going over kind of different scenarios like if you can purchase this much or just different foods that they can make recipes along with the food and vegetables. So trying to encourage families to use all of the full amount of the fruit and vegetable." – Local agency director (mostly urban, less than 750 caseload, ITO/Territory)<br><br>"We did mail out, so we mailed something like a flyer that had that information to each of our WIC families. So we mailed something out. Again, it was on

			more information on how to use the increased amount.	our social media platforms. Whenever I would print off a list of families' benefits, I would circle or highlight that new or additional amount. When doing outreach, that was a huge talking point when kind of promoting WIC." – Local agency director (mostly rural, less than 750 caseload)

Table 6. Caregivers' experience with the various cash value benefit (CVB) amounts

CVB Amount	Theme	Description	Quote
$9/Child/Month<br><br>Average Satisfaction Score (out of 10): 5.3	Caregivers felt the $9/child/month CVB was not sufficient to provide an adequate amount and variety of fruits and vegetables for children.	In general, caregivers mentioned utilizing the CVB each month; however, they noted the $9/child/month CVB amount was used within the first two shopping trips of the month. Caregivers typically used money out of pocket to cover extra fruits or vegetables they provided for their children. In addition, with the $9 CVB, caregivers gravitated towards more affordable and limited selection of fruits and vegetables such as bananas, apples, and carrots for their children, often limiting the variety of fruits and vegetables children were exposed to.	"I wasn't really satisfied because it [the CVB] really wasn't much because my little girl, when she was on it, like I said, she loved vegetables and fruits and my little baby, he loved fruits. He like apples and oranges. So, the $9 wasn't nothing with two, and $9 really not much for one due to the vegetables and stuff and the fruits [price] went up a lot."<br><br>"With the $9 that would go in pretty much one trip with, I would try to buy the cheaper fruits with the WIC, so the bananas where it's only a certain amount of cents per pound rather than the expensive or berries and things like that to stretch that $9 as far as I could. But yeah, I made sure that we used the entirety of it each month."
$35/Child/Month<br><br>Average Satisfaction Score (out of 10): 9.8	Caregivers felt using the $35/child/month CVB was beneficial to expand or supplement their household finances.	Many caregivers reported using the full CVB amount each month and noted that the CVB amount lasted longer so they could purchase fruits and vegetables throughout the month. Reduced out of pocket grocery expenses, fewer household impacts of inflation, and less concern that they would not meet their household grocery needs were other advantages caregivers noted with the $35/child/month CVB.	"Oh yeah, like triple the amount I was able to, well, I make sure I pay attention to sales and prices because that's what I'm used to with having to work with the $9. And so with that I'm able, I can spend $15 this time and $15 next time, maybe $10 more. I'm able to split it up and be able to get my kids fresh fruit and veggies on a weekly basis instead of trying to make salad last or apples last a month..."

			"I wasn't worrying whether I was going to have enough [money]. It really made a difference to be able to go into a store and think, you know what, I'm going to be able to get what I came here for. It's not just going to be, you can only get so much of this or so much of that."
	The higher CVB amount allowed caregivers to purchase an increased variety and amount of fruits and vegetables for their household.	Several caregivers reported that they purchased wider varieties of fruits and vegetables (e.g., mango, pomegranate, berries) for their household compared to their usual purchases when the CVB amount was lower. Freedom to purchase produce in larger amounts and introducing new varieties to their children were commonly mentioned, and caregivers felt the CVB amount allowed them to purchase things their household wanted rather than only what they needed.	"I felt like I had a little bit more freedom to buy maybe different vegetables that were higher priced. I felt like more comfortable buying a bag of grapes that are typically like $8 a bag now. Just buying a bag of grapes was at one point it was like that's all of the benefits. And then with the increase it was like I could get that and then some more. So I felt like it offered the household more freedom to just have a variety of maybe more expensive fruit and vegetables."<br><br>"So yeah, I'd get kind of the same fruit, but then I was able to be able to afford a bag of oranges and that makes a huge difference to my kids. And then I used a good portion of it for vegetables and that was huge too, because it just helped us eat correctly, even more so than before. I make sure I include mostly veggies with every meal now, and it's a huge difference."
$24/$25/Child/Month<br><br>Average Satisfaction Score (out of 10): 7.8	Caregivers implemented budgeting strategies and were more selective in the fruits and vegetables they	While caregivers mentioned they were still grateful for receiving the $24/$25/child/month CVB, they noted being more selective in the fruits and vegetables they	"It probably just made me a little more aware. Again, it [the CVB] wasn't a huge cut. It didn't go all the way back down to $9, so it wasn't enough where I was like, 'Oh, I need to really cut back.'

	purchased when the CVB decreased.	were purchasing compared to the $35/child/month CVB. Many caregivers reported the price of fruits and vegetables had increased during this time, so they were carefully monitoring the CVB they were using and choosing items that were on sale or in season when the CVB decreased. In some situations, caregivers were using their own funds to supplement what was still needed to purchase fruits and vegetables.	But I think it was more that I was just like, 'Okay, I don't have as much, so I'm going to just maybe be a little bit more particular and making sure.' Because sometimes it's hard with produce if you buy stuff and then you don't eat it in time, it can go bad. So, I would just probably try to be a little more careful so that we are using everything for sure, that we're getting, since we had a little less."<br><br>"Well, I mean obviously it [the CVB] is less, so I'm kind of going back into that really having to logically think out veggie planning rather…So, I definitely think that we've been eating less vegetables in the household."
	When the CVB amount decreased, caregivers reported a change in the variety of fruits and vegetables they were purchasing.	Caregivers found themselves returning to more familiar fruits and vegetables (e.g., apples, bananas, carrots) they knew their children would eat when the CVB amount decreased. With the decrease in the dollar amount of the CVB, caregivers tended to be more selective in the types of produce they provided their children to ensure it would not go to waste. Similarly, caregivers mentioned selecting more frozen options of fruits and vegetables compared to fresh since they knew it would last longer.	"...I did like the $35, so I could try to keep introducing different types, especially the vegetables, trying to encourage them to eat more vegetables. I would try to do that a lot more often with the $35. Now that it's lower, I don't really try as much because I know that if they don't eat it, then I feel like it's just going to waste, whereas I know they'll eat the fruits."<br><br>"Well, compared to the $35, it did make me kind of more conscientious about how many varieties I'm getting at once and kind of waiting until all of the fruits and vegetables in the house actually ran

			out before going to get any more just to make sure that it would last throughout the whole month."

Table 7. Caregivers' reported facilitators and challenges utilizing the cash-value benefit (CVB)

Theme	Description	Quote
Caregivers relied on various modes of information and a variety of resources to facilitate use and awareness of the CVB.	Most caregivers received information about the CVB through WIC appointments. When the CVB amount changed from $35 to $24/child/month, caregivers said staff members would inform them prior to the benefit amount changing so they were able to plan ahead and utilize the higher benefit amount. Additionally, caregivers in states that had the ability to send out mass texting or social media blasts noted learning of the change ahead of time through those communication efforts. For resources that helped caregivers utilize the CVB, one resource notably mentioned was the WICShopper App or any application ran by the State to help identify which fruits and vegetables could be purchased in store.	"Because when I go to recertify for my WIC, the lady always tell me, she always tell me, 'It went down this amount, it went down that amount.' So she kind of tell me the day that I go, when I go pick up. So she kind of give me a heads-up to let me know so I won't be go to the store thinking I got this certain amount because I'm so used to this certain amount. So she give me, like I said, she give me heads up, 'Well, you getting this amount of that amount this month."<br><br>"I would say pretty much when it happened. Like I said, our local agency is really good about letting people know the changes that happen to their food packages as well as the WICShopper App. Being able to look at what benefits I have on my card for this month and next month, that helps too."
Some caregivers were unaware the CVB changed and faced challenges during store checkout.	Caregivers expressed frustration and challenges around not knowing the CVB had changed. Some caregivers were unaware the CVB increased to $35/child/month since they were not notified by their WIC office, and similarly when the amount changed to $24/$25/child/month. For some, they only found out about the change in the CVB when they attempted to utilize the previously higher CVB amount issued at the store. Thus, resulting in caregivers not able to purchase the fruits and vegetables they originally	"I don't think it was until I went to go shop and hopped on the app and was seeing... Okay. Even though I pretty much know by heart what we're usually getting, but it changed and so it wasn't until that time I realized that it went back down and so it was a little disheartening because I realized I can't get the normal stuff we would like. I couldn't get, if we wanted bell peppers, I probably couldn't have got that, just because even though it was a small cut, it

	planned to, or having to pay the out-of-pocket difference in line.	still made a difference to us with the prices of everything. It's expensive, it's so expensive right now."<br><br>"I found out when we were standing in the line…So I kind of know what our total is going to be within, honestly, $25. And so when it was pushing like $35 or $45 more than what I originally saw, that's when I was like, 'Well, what's going on?' And she [the cashier] was like, 'Well, this is what was covered.' And that's when she printed out that first receipt that tells you what's covered."
Caregivers expressed frustration around access and availability of affordable, WIC-eligible items when utilizing the CVB in store, exacerbated by COVID-related impacts.	One major challenge caregivers experienced when trying to use the CVB was certain fruits and vegetables not registering at the store, even though they were WIC-approved items. With the CVB increase coming during the height of the pandemic, caregivers experienced additional issues with availability and acceptability of produce at grocery stores and sometimes had to travel to various stores to find appropriate items. Other barriers to using the CVB included variable grocery store hours that limited the time caregivers could shop, increased risk of COVID with in-person shopping, and CVB restrictions on what type of produce (e.g., fresh, frozen, canned) could be purchased.	"Well, some of the stores that I went to didn't actually accept the WIC funds, so I had to shop most of my products from [STORE]. And the distance from where I'm staying to the [STORE] nearby somehow a long distance other than the stores that were readily available. So I had to go all the way to [STORE] just for me to... In order for me to access the funds on the WIC program."<br><br>"I have noticed that there can be some inconsistencies from week to week, like I'll run out of let's say frozen Brussels sprouts, and so the next time I'm at the store I'll get the exact same brand, exact same size, and everything, and then it'll register as not being an approved item, and it's like, 'I just bought this last week. I know it works.'"

Table 8. Caregivers' overall perceptions of using the cash-value benefit (CVB)

Theme	Description	Quote
Caregivers reported using the CVB provided both financial and dietary advantages for their children and their household.	Providing fruits and vegetables to their children is important to caregivers and using the CVB allows them to have more financial power to do so. With fewer concerns about being able to afford groceries, caregivers noted that they can serve fruits and vegetables to their children more often, reinforce their nutrition education, and maintain a more healthful diet overall.	"Not having to stress out about if I have enough in the house. Like I said, this is an added, the fruits and veggies money is something added on to what I'm already spending. Don't have to worry, 'Well, do I buy this or do I cut back on this?' I don't have to worry about that now I can just get it if I need it."<br><br>"I would say that it's made my kids a lot more happier because they're able to have fruits and vegetables with every single meal rather than when it was just the $9. It was mainly just with breakfast and sometimes with lunch. So I mean, the amount being higher, it's made a huge difference in the sense that they get fruits and vegetables with every single meal, and when they have snacks, they can go grab apples or oranges or anything like that and have more healthy options versus unhealthy."
Decreasing the CVB amount back to $9/child/month would create challenges for caregivers to continue providing a nutrient rich diet for their children.	Caregivers noted that rising food costs and a higher cost of living would not allow the $9 CVB to stretch as far, and it would be difficult to continue offering their children fruits and vegetables in the same capacity if the CVB decreased. Some noted that they would have to return to a stricter household budget and reduce the variety and frequency of fruits and vegetables they give their children.	"I'd probably be upset. I'd deal with it because I know there's kind of no other option, but I'd be really bummed. It'd be hard to provide the fresh stuff my kids need, because in reality that's what they eat the most. They really do."<br><br>"I'd be really sad. I'd be really devastated that changes everything because you can barely... I mean, with the price increases

		now of just food in general and, I mean, everything's went up, we would be lucky if that would last us a week to two weeks if I did it for just the kids that get WIC even, I mean, limited to two of the kids that are on WIC, it would maybe last us two weeks."
The average ideal CVB amount reported by caregivers was $49/child/month, with a range from $25 to $80.	The common ideal benefit amount ranged from $25-$80/child/month, as caregivers felt this was appropriate to purchase everything they need considering current food costs. Some noted that increasing the CVB back to $35 would be sufficient. Participants in more urban areas were more likely to suggest $50/child/month or more.	"To not fully pay for it, but definitely help supplement and make sure that we were doing a bare minimum of providing some really healthy options, $10 a week. So maybe $40 to $50 a month I think would be more than enough to really, really ensure that every week they were getting some fresh things."<br><br>"That's a good question, 'cause it does seem like things are more expensive. I mean, a rough estimate maybe would be $10 a week. I mean, probably more. Who knows? It's so hard to tell. Maybe more, like $10 to $15 a week. So, $40 to $60 a month I guess, to cover the full amount. "
Most caregivers reported they would still participate in WIC if the CVB were to decrease but noted their level of participation and satisfaction with the program would likely decrease.	Though most caregivers felt the other benefits and services WIC provides are helpful, the CVB was highlighted as a key reason for participating in the program. Some mentioned that they would have to rely on other assistance (i.e., SNAP) more heavily and that they would be less likely to keep up their certification with the smaller value of the CVB. Those that noted that the CVB is the only thing they use from WIC felt they would likely not continue in the program.	"The biggest reason I'm probably still on WIC is because of the fruit and veggies. I started getting on there for the formula, but this time around, I'm a breastfeeding mom...I'm not formula feeding, so that's kind of why it took me so long to even use my benefits when I started getting them is, 'Well, I don't need formula, so why even mess with this WIC shopping?' But the fruits and veggies, yeah, that's worth staying on WIC and being on WIC, really."

		"I would still be participating even if it [the CVB] wasn't a part of it. It's just something that has made it a lot better. And I know before there's been times that I've actually forgot to re-certify or go in and do everything like that, but having that on there now, it's like, 'Oh, I know better than to miss my appointments.'"

Table 9. Barriers to cash-value benefit (CVB) redemption among caregivers who identify as Black, Indigenous, and People of Color (BIPOC) (n=46)

BIPOC Caregiver Barriers	Description	Quotes
Limited food access	The impacts of hurricanes in Puerto Rico limited the availability of fresh produce in stores, particularly when the CVB amount was $9.	"You couldn't get the products that you were looking for, based on the season of each product. Like the banana, you couldn't get those because of the pandemic, or before because of the hurricane, you couldn't get them."– Hispanic Black participant
Lack of CVB change awareness	There were various instances where BIPOC participants discussed not being aware of the CVB changes or the foods that could be purchased with the benefit:<br>• Many participants in Puerto Rico and some Native American participants were not aware of the increase to $35 and noted that they had to call their WIC office to get information about the CVB.<br>• Many participants of various ethnicities in rural areas were not aware of the change from $35 to $24 and claimed they were not notified by their WIC office. These participants most often found out about the change by looking at their benefits through their state app, during checkout at the grocery store, or through social media.<br>• Two Black participants in urban areas were unaware that they could purchase canned and frozen varieties of fruits and vegetables.	"I think it was in the news, no wait, on their Facebook page. They put this in the news because they were already telling people that it was going to change. And then they put all the information for, the amount for nursing mothers is this much, the children is this much, they divided it like that…Since I follow WIC social pages, I'm a little more aware of the news. I think, when I went there, I found out. Other people who sometimes cannot do that, maybe they did not know." – Hispanic White participant
Limited WIC-authorized vendor access	In some rural areas, Native American participants noted they could only access one or	"Being in a rural area, I mean, we're not quite food desert, but we're pretty close. There's the closest big grocery store

	two nearby stores, and stock was inconsistent. This was the case both when the CVB was $9 and when it increased to $35.	like a [RETAILER] is a 15, 20 minute drive away, and there's a little market here in town, but they almost never have anything besides prepackaged salad mixes. So fresh fruits and vegetables were something that were just it was not practical for us. We needed things that would travel well and would keep well, because we couldn't afford to go to the store every week, especially with gas prices what they are." – Native American participant<br><br>"During that time, at least in my area, things were kind of limited at grocery stores. And so there was sometimes that it was hard to use just because of what was available. There would be like apples left were not ones you would want to eat. And things like that would happen a lot." – Native American participant
Increased food expenses	The cost of produce in Puerto Rico made it difficult for participants to purchase enough produce for their family with the $25 amount, and they often had to use their own money to cover the extra costs.	"So, since the pandemic happened, the availability of fruits and vegetables has been limited. Well, now, here in Puerto Rico, Hurricane Fiona happened. So, now fruits are very scarce and expensive." – Hispanic White participant

Table 10. Barriers to cash-value benefit (CVB) implementation among agencies with high racial diversity (n=30)

Local Agency Barriers	Description	Quotes
Excessive implementation workload (e.g., process, timing, guidance)	Local agencies, specifically among those with higher caseloads, noted some caregivers did not receive the higher benefit amounts when they were issued. In some cases, local agencies had WIC participants physically come into the clinic to receive the correct benefit amounts, which overwhelmed staff. Reports of not having adequate time, staff allocation, or resources to issue the food package were causes of participants not receiving higher CVB amounts.<br><br>Additionally, staff mentioned feeling the burden of having to manually issue benefits, often taking time away from time allocated to appointments and everyday clinic duties.	"We did have to go back and [void and reissue] a lot of food packages, which was unfortunate because we have so many patients that even though the state was providing us a list of all the [clients], we were not able to. First of all, [the list] wasn't very accurate, and second of all, we were not able to change it by the time [benefits were issued]. So a lot of people had to call to get this change in their food package. […]it takes a long time when it's over 6,000 people or so. So yeah, if they found a way to implement it without us having to even intervene at all, maybe our only intervention would be to educate people, that would be great." – WIC local agency staff<br><br>"[The participants] have to bring the card. […] all our clients have to come in. So they would call me and say, 'Did [the CVB amount] go up?' And I'd say, 'Yes.' Then they'd have to come in and I'd void and reissue two months worth or whatever, whatever months they didn't get the higher amount." – WIC local agency staff (offline state)

Difficulties with promotion/marketing	Local agency staff worked to promote the CVB changes through various modes of communication (i.e., texting, emails, flyers, newsletters, and social media). Often, the responsibility to ensure the right amounts were allocated fell on WIC participants, who were encouraged to call or check in with their WIC staff during their appointments. As a result, WIC staff were fielding more calls from participants and had to be available to answer a variety of questions about the CVB increases. Some participants were unable to receive updates due to not having a phone or a reliable phone number or not checking their mail frequently.	"During COVID, when we were closed for that long period of time, a lot of our clients, their telephone numbers come and go a lot, so we went back to old-fashioned mailing." – WIC local agency staff
Resources and staffing	Local agency staff were the ones in charge of manually issuing benefits when clinics could not automatically implement the CVB changes. Larger local agencies were often able to allocate a large number of staff members to these tasks; however, for smaller local agencies or agencies dealing with staffing shortages, they noted the burden often fell on a few staff members to make the change. As a result, clinic duties and staff time had to be reallocated (i.e., less time for appointments).	"Due to staffing and capacity, we were not able to reach out to every single person. Really, the benefits showed up for them and they would see that increased dollar amount. So, anyone that we were encountering, we were telling them. But if it was a void and reissue, they just got their benefits. Some clinics tried when they re-tailored to send a text and let them know, but not everybody even had the staffing to be able to do that." – WIC local agency staff
